# Molecular and behavioral consequences of *Ube3a* gene overdosage in mice

**DOI:** 10.1172/jci.insight.158953

**Published:** 2022-09-22

**Authors:** A. Mattijs Punt, Matthew C. Judson, Michael S. Sidorov, Brittany N. Williams, Naomi S. Johnson, Sabine Belder, Dion den Hertog, Courtney R. Davis, Maximillian S. Feygin, Patrick F. Lang, Mehrnoush Aghadavoud Jolfaei, Patrick J. Curran, Wilfred F.J. van IJcken, Ype Elgersma, Benjamin D. Philpot

**Affiliations:** 1 Department of Clinical Genetics and Department of Neuroscience and; 2 ENCORE Expertise Center for Neurodevelopmental Disorders, Erasmus MC, Rotterdam, Netherlands.; 3 Neuroscience Center, Department of Cell Biology and Physiology, and the Carolina Institute for Developmental Disabilities and; 4 Department of Psychology and Neuroscience, University of North Carolina at Chapel Hill, Chapel Hill, North Carolina, USA.; 5 Center for Biomics, Erasmus MC, Rotterdam, Netherlands.

**Keywords:** Neuroscience, Behavior, Mouse models, Neurological disorders

## Abstract

Chromosome 15q11.2–q13.1 duplication syndrome (Dup15q syndrome) is a severe neurodevelopmental disorder characterized by intellectual disability, impaired motor coordination, and autism spectrum disorder. Chromosomal multiplication of the *UBE3A* gene is presumed to be the primary driver of Dup15q pathophysiology, given that *UBE3A* exhibits maternal monoallelic expression in neurons and that maternal duplications typically yield far more severe neurodevelopmental outcomes than paternal duplications. However, studies into the pathogenic effects of UBE3A overexpression in mice have yielded conflicting results. Here, we investigated the neurodevelopmental impact of *Ube3a* gene overdosage using bacterial artificial chromosome–based transgenic mouse models (*Ube3a^OE^*) that recapitulate the increases in *Ube3a* copy number most often observed in Dup15q. In contrast to previously published *Ube3a* overexpression models, *Ube3a^OE^* mice were indistinguishable from wild-type controls on a number of molecular and behavioral measures, despite suffering increased mortality when challenged with seizures, a phenotype reminiscent of sudden unexpected death in epilepsy. Collectively, our data support a model wherein pathogenic synergy between *UBE3A* and other overexpressed 15q11.2–q13.1 genes is required for full penetrance of Dup15q syndrome phenotypes.

## Introduction

Human chromosome 15q11.2–q13.1 is exceptionally vulnerable to structural abnormalities that result in neurological disorders (1–5). Clusters of repetitive sequence, which originated in part from duplications of the GOLGA8 gene, bring about the existence of 5 distinct breakpoint sites (BP1–BP5) spanning this region (6–11). These breakpoints increase the risk of homologous recombination during meiosis, resulting in either deletions or duplications of 15q11.2–q13.1 (4, 6, 11, 12).

Genomic imprinting underlies the monoallelic, parent-of-origin–specific expression of different 15q11.2–q13.1 genes. Consequently, paternal and maternal 15q11.2–q13.1 deletions produce distinct pathophysiologies, which in turn result in Prader-Willi and Angelman syndromes, respectively (13, 14). Maternal duplications of this same region are deemed to be causative of a neuropsychiatric disorder called Dup15q syndrome (11, 15). Dup15q syndrome is clinically defined by moderate to profound intellectual disability, impaired motor coordination, and autism spectrum disorder (ASD) (16–18). Some patients may present with interstitial duplications, a condition referred to as a 15q11.2–q13.1 trisomy. However, in the majority of the duplication events, BP1–BP3 recombination results in an isodicentric triplication of the 15q11.2–q13.1 region [idic(15)], giving rise to a supernumerary chromosome 15 or a 15q11.2–q13.1 tetrasomy (11, 19). Dup15q syndrome pathological severity increases proportionally with the number of 15q11.2–q13.1 copies, meaning that idic(15) individuals, in general, have more severe symptomology than individuals with interstitial duplications (16, 19).

Efforts to elucidate the pathophysiological contributions of specific genes to Dup15q syndrome have focused on *UBE3A* (20–22). Of all the genes in the 15q11.2–q13.1 region, *UBE3A* alone exhibits cell type–specific, maternal monoallelic expression. The paternal *UBE3A* allele is silenced in mature neurons, leaving maternal *UBE3A* as the sole source of UBE3A protein in these cells (23–25). Thus, neuronal UBE3A overdosage is unique to maternally inherited 15q11.2–q13.1 duplications, which yield far more severe neurodevelopmental phenotypes as compared with those of paternal origin (11, 15, 26). Additionally, maternal inheritance of a circumscribed *UBE3A* gene duplication has been linked to developmental delay and neuropsychiatric phenotypes in multiple members of a single family; family members with paternal inheritance of the same mutation were unaffected (27). Such findings have focused the lens on *UBE3A* gene duplications as being a major driver of disease pathology in Dup15q syndrome.

*UBE3A* encodes a HECT E3 ubiquitin ligase involved in ubiquitin-mediated protein turnover (28, 29). It is commonly believed that the ability of UBE3A to control the abundance of its protein substrates is imperative to prevent disease (30, 31). UBE3A is also strongly implicated in transcriptional coactivation (32–35), another function that may be critical to maintaining cellular homeostasis. Although UBE3A deficiency indisputably leads to Angelman syndrome (14), a causal connection between UBE3A overexpression and Dup15q syndrome phenotypes has proved elusive. Not only is clinical evidence of *UBE3A* microduplication sparse (27), but also studies of the consequences of UBE3A overexpression in mouse models are contradictory. In 2009, Nakatani and colleagues reported on mice harboring a duplication of chromosome 7, the syntenic 15q11.2–q13.1 region in mice. Surprisingly, it was mice with paternal duplication that showed ASD-like phenotypes in this study. Mice with maternal duplication showed no notable behavioral abnormalities, challenging expectations based on maternal inheritance of Dup15q syndrome (36). In later research, various groups homed in on UBE3A overexpression alone. These efforts yielded novel transgenic mice that were shown to display phenotypes reminiscent of Dup15q syndrome pathology (20–22). However, design features inherent to these models — overly excessive *Ube3a* copy number (21, 22), homozygous inheritance of transgenic alleles (20, 22), restricted UBE3A isoform representation (21), and the incorporation of function-altering protein tags (20–22) — have confounded interpretations of their pathophysiological relevance to Dup15q syndrome.

In this study we describe transgenic mice for modeling UBE3A overdosage as it would most likely occur in Dup15q syndrome. Our model prioritizes disease-relevant excess of *Ube3a* gene copies, the full representation of enzymatically competent UBE3A isoforms, and the faithful recapitulation of endogenous UBE3A expression patterns in the brain. By rigorously testing these mice for changes in gene expression, synaptic physiology, and performance in UBE3A-sensitive and Dup15q-relevant behavioral assays, we revisit UBE3A’s contribution to Dup15q syndrome pathophysiology from a position of improved construct validity.

## Results

### Generation and validation of the Ube3a^OE^ mouse model.

**Figure 1:**
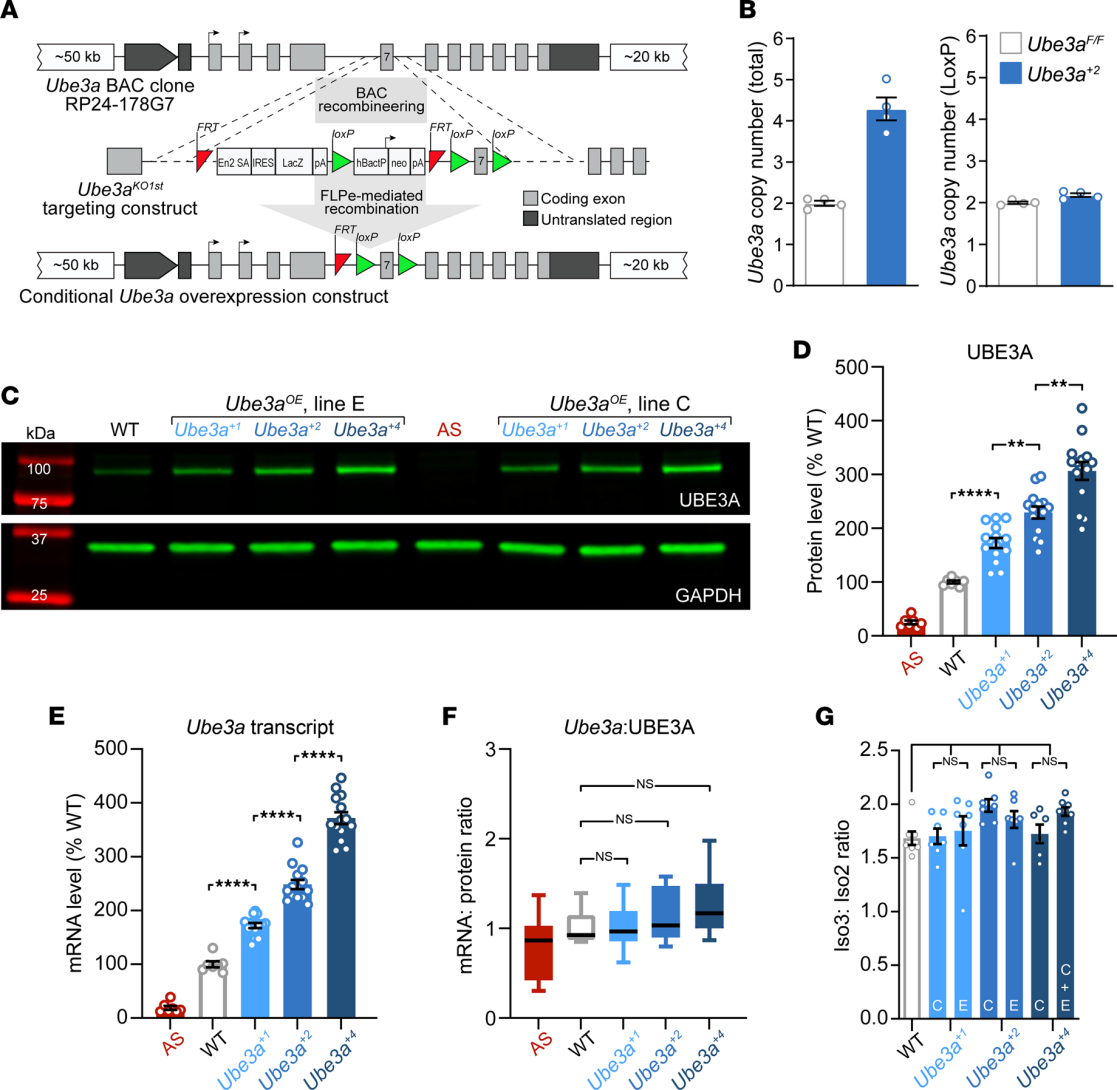
Generation and validation of *Ube3a^OE^* mice with dose-dependent overexpression of *Ube3a* transcript and UBE3A protein. (**A**) Schematic of BAC transgenic strategy to generate conditional *Ube3a* overexpression mice. Exon/intron numbering is relative to *Ube3a* isoform 2. (**B**) Droplet digital PCR (ddPCR) analysis of total and transgenic *Ube3a* genomic copy number in *Ube3a^+2^* overexpression mice from line E. ddPCR assays specific to the *Ube3a* intron 3/exon 3 boundary were used to assess total (i.e., endogenous plus transgenic) *Ube3a* copy number (left); ddPCR amplification of *loxP* sequences enabled specific detection of transgenic *Ube3a* copies (right). Homozygous floxed *Ube3a*-knockin mice (*Ube3a^fl/fl^*) served as controls. (**C**) Representative Western blot (WB) depicting immunofluorescent detection of bands corresponding to total whole-brain UBE3A and the loading control protein, GAPDH. Bands at left (red) correspond to the molecular weight marker. (**D**) Mean ± SEM UBE3A WB immunofluorescence intensities as determined from WT (*n* = 7), AS (*n* = 7), and *Ube3a^+1^* (*n* = 14), *Ube3a^+2^* (*n* = 14), and *Ube3a^+4^* (*n* = 14) whole-brain lysates. Welch’s ANOVA, Dunnett’s post hoc. (**E**) Mean ± SEM whole-brain *Ube3a* transcript levels as determined from ddPCR experiments (2.5 ng cDNA input). Samples were prepared from cerebral hemispheres opposite those used to prepare the protein lysates assayed in **D**. One-way ANOVA, Tukey’s post hoc. (**F**) Box plots of ratios of whole-brain *Ube3a* transcript and UBE3A protein levels for corresponding samples from **E** and **D**, respectively. Whiskers represent 5% to 95% confidence intervals. One-way ANOVA. (**G**) Mean ± SEM ratios of *Ube3a* isoform 3 to *Ube3a* isoform 2 transcript levels, broken out by specific *Ube3a^OE^* lines, as determined from ddPCR experiments. One-way ANOVA. **P < 0.01, ****P < 0.0001.

We used a bacterial artificial chromosome (BAC) transgenic approach to generate potentially novel mouse models of UBE3A overexpression, henceforth referred to as *Ube3a^OE^* mice. Seeking a transgene encoding enzymatically competent UBE3A protein, capable of expressing both UBE3A isoforms according to endogenous spatiotemporal patterns, we chose as our starting point a BAC clone encompassing the entire wild-type (WT) mouse *Ube3a* coding region as well as large stretches of flanking, untranslated sequence both upstream (~50 kb) and downstream (~20 kb). Subsequent recombineering and flippase-mediated (FLP-mediated) recombination in *E. coli* culminated in a floxed construct with the capacity for Cre-mediated cessation of expression (Figure 1A). Our efforts yielded 6 independent *Ube3a^OE^* mouse lines (lines A–F), 2 of which (lines C and E) proved to harbor transgene insertions within chromosome 3, as determined by targeted locus amplification mapping using sequencing primer sets designed to provide coverage of *Ube3a* and nearby flanking sequences (Supplemental Tables 1 and 2; supplemental material available online with this article; https://doi.org/10.1172/jci.insight.158953DS1). Using complementary droplet digital PCR (ddPCR) assays targeting both endogenous and *loxP*-containing genomic DNA sequences, we further determined that each line carried a 2-copy transgenic *Ube3a* insertion (Figure 1B, Supplemental Figure 1A, and Supplemental Table 3). These 2-copy *Ube3a^OE^* lines, termed *Ube3a^+2^*, mimic the *UBE3A* gene overdosage observed in idic(15) individuals. *Ube3a^OE^* transgene copies contain a residual FRT site, a by-product of our recombineering strategy (Figure 1A). This presented an opportunity to generate 1-copy *Ube3a^OE^* sublines of mice (*Ube3a^+1^*) with high construct validity for *UBE3A* overdosage in interstitial Dup15q, which we exploited by crossing *Ube3a^+2^* mice to a highly efficient FLPo deleter line (37) (Supplemental Figure 1B). Follow-up genomic ddPCR experiments verified the efficacy of this approach for both line C and line E mice (Supplemental Figure 1, C and D).

We next determined the extent to which *Ube3a^OE^* mice overexpress UBE3A protein in the brain, using a Western blotting approach calibrated for linear detection of increased UBE3A protein content at least 2-fold above WT levels within whole-brain protein lysates (Supplemental Figure 2A). Given that the paternal *Ube3a* allele is epigenetically silenced in neurons (23–25), WT control mice express 1 functional neuronal copy of *Ube3a*, whereas *Ube3a^+2^* mice express 3 copies. Assuming perfectly additive UBE3A protein expression with each stepwise increase in *Ube3a* gene copy number, *Ube3a^+1^*, *Ube3a^+2^*, and *Ube3a^+4^* (produced from *Ube3a^+2^* × *Ube3a^+2^* matings) mice should overexpress UBE3A 1-fold, 2-fold, and 4-fold, respectively (i.e., 200%, 300%, and 500% of WT). In fact, we observed more modest percentage gains in UBE3A protein expression relative to WT: *Ube3a^+1^* = 172.5% ± 9.23%; *Ube3a^+2^* = 229.3% ± 11.01%; *Ube3a^+4^* = 306% ± 16.7% (Figure 1, C and D). This relationship was equally evident in line C and line E *Ube3a^OE^* samples (Figure 1D and Supplemental Figure 2B). Leveraging a reverse transcriptase ddPCR assay with a dynamic linear range encompassing many-fold increases in WT *Ube3a* expression (Supplemental Figure 2C), we found a relationship between increasing *Ube3a* gene copy number and accumulating transcript level (Figure 1E and Supplemental Figure 2D) that mirrored our findings for UBE3A protein (Figure 1, C and D, and Supplemental Figure 2B). Accordingly, within-animal ratios of *Ube3a* transcript and UBE3A protein were near 1 and proved statistically indistinguishable by genotype (Figure 1F and Supplemental Figure 2E). Extensive follow-up ddPCR studies showed that increasing *Ube3a* dose does not affect *Ube3a* isoform ratios (Figure 1G and Supplemental Figure 2, F and G). Together, these data suggest limits to gene dose–dependent increases in UBE3A expression that are largely imposed at a transcriptional level, irrespective of *Ube3a* isoform.

**Figure 2:**
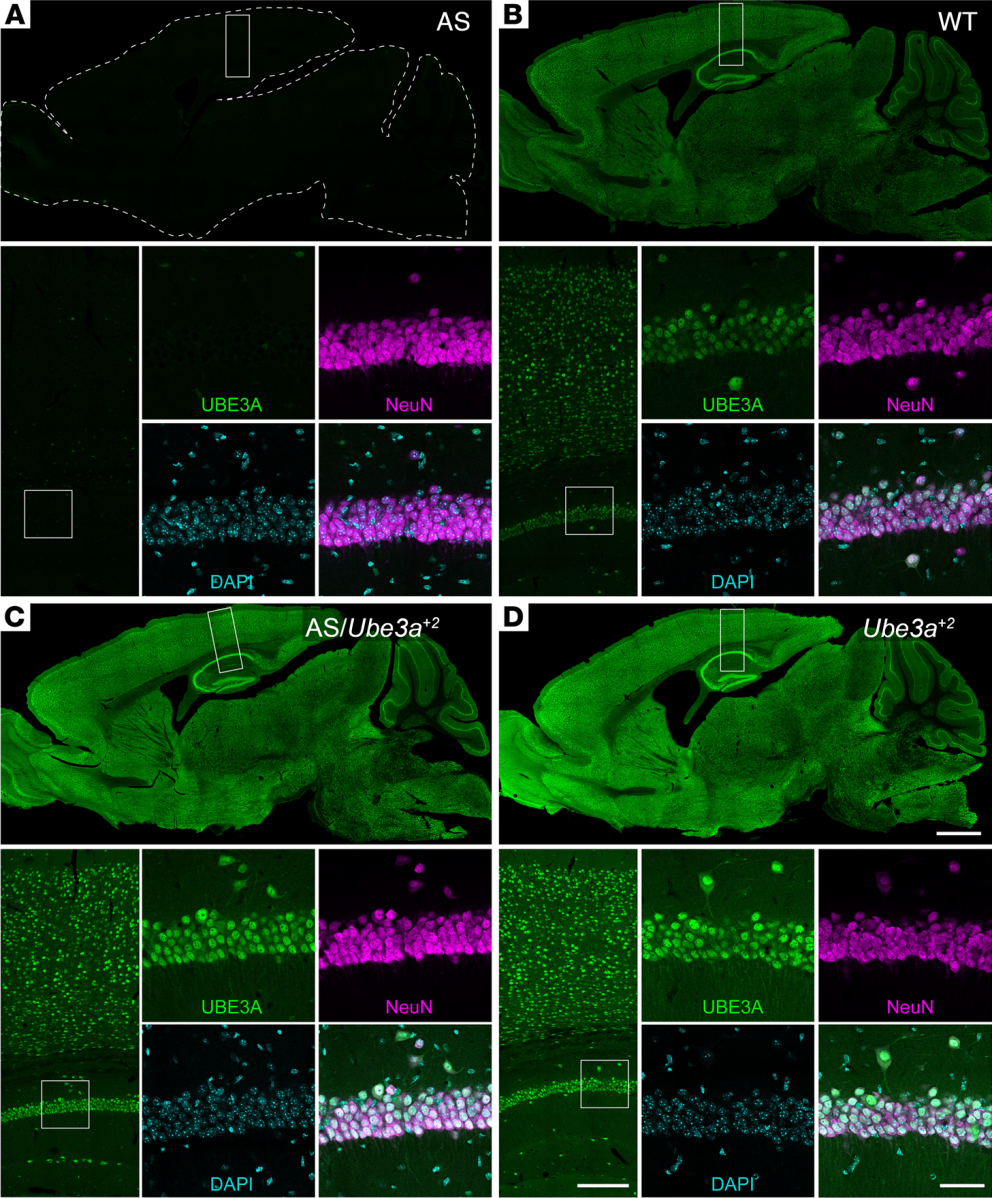
*Ube3a^OE^* mice express transgenic UBE3A protein according to endogenous patterns. (**A**–**D**) UBE3A immunofluorescence staining (green) in sagittal brain sections from postnatal day 25 (P25) AS (**A**), WT (**B**), AS/*Ube3a^+2^* double-mutant (**C**), and *Ube3a^+2^* mice (**D**). Sections are counterstained with NeuN (magenta) and DAPI (cyan). Boxes indicate regions of interest for higher-magnification images shown in successive panels. Scale bars: 1 mm, 200 μm, 50 μm.

Having established gene dose–dependent dynamics of *Ube3a* transcript and UBE3A protein overexpression, we carried out subsequent *Ube3a^OE^* characterization experiments, focusing on mice from line E. Here, we first sought to evaluate transgenic UBE3A protein biodistribution in the brain. To facilitate these experiments, we crossed *Ube3a^+2^* mice to Angelman syndrome (AS) model mice (AS/*Ube3a^+2^*), as the latter are devoid of UBE3A expression in mature neurons (38), effectively providing a blank backdrop against which *Ube3a^OE^* transgene expression can be plainly observed (Figure 2A). UBE3A immunofluorescence appeared to be elevated in the brains of AS/*Ube3a^+2^* mice, and even more so in *Ube3a^+2^* single mutants, but the spatial distribution of this signal did not differ from that in WT controls: all 3 groups exhibited virtually ubiquitous UBE3A labeling in neurons throughout the brain. Inspection of individual neurons revealed similarly well-conserved UBE3A protein distribution at the subcellular level (Figure 2, B–D), characterized by a pronounced concentration in the nucleus, which is appropriate for the fourth postnatal week of brain development and beyond in mice (39, 40). AS/*Ube3a^+2^* mice also displayed age-appropriate UBE3A distribution in the early postnatal brain, matching WT control mice with regard to transient enrichment in striatal patches and laminar patterning in the neocortex (Supplemental Figure 3). Thus, endogenous UBE3A expression patterns are faithfully recapitulated in *Ube3a^+2^* mice.

**Figure 3:**
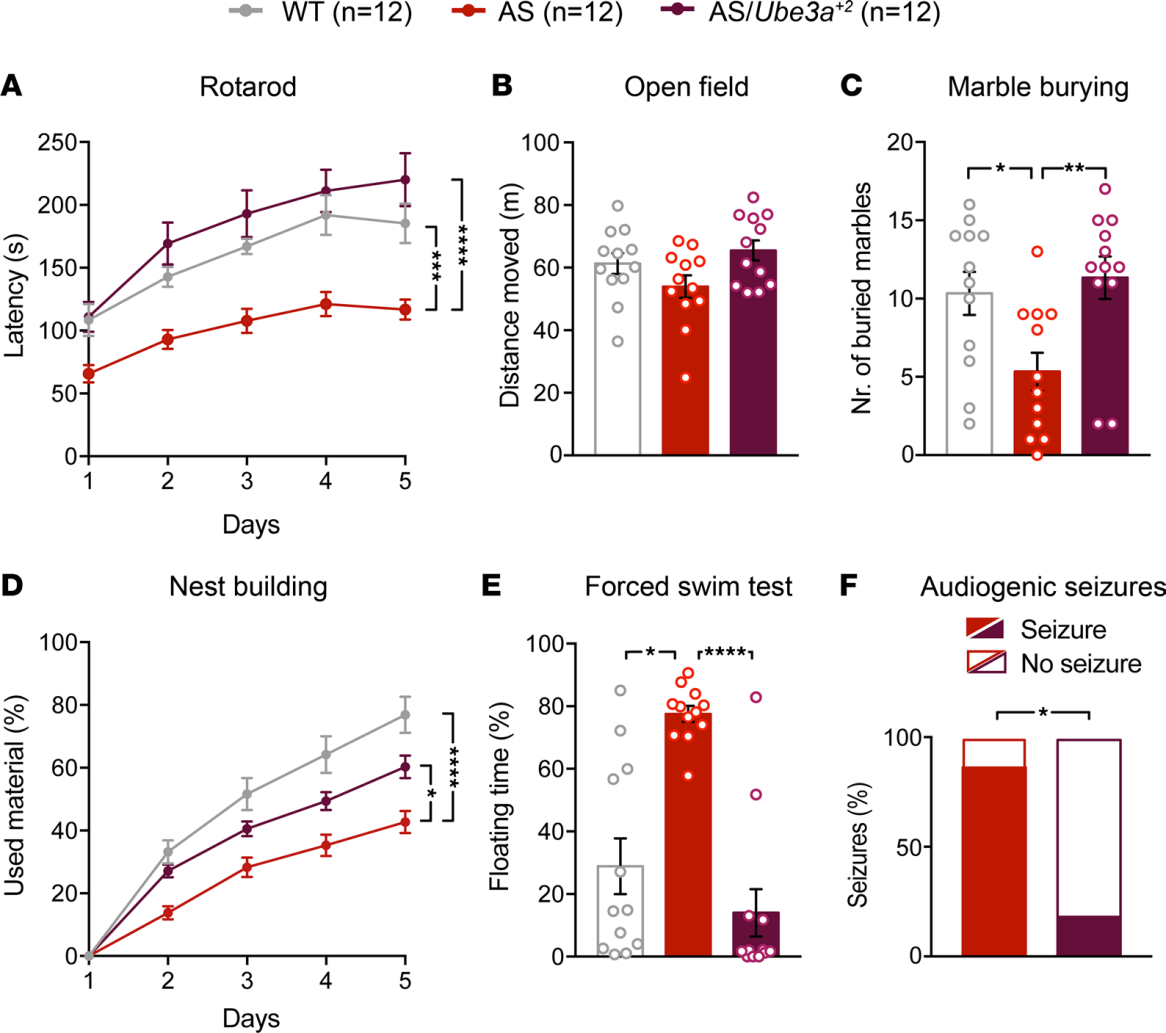
Transgenic UBE3A protein rescues behavioral phenotypes observed in AS mice. (**A**–**F**) Mean ± SEM group performance of WT, *Ube3a^m–/p+^* (AS), and *Ube3a^m–/p+^* × *Ube3a^+2^* (AS/*Ube3a^+2^*) mice for fall latency on the reversed rotarod task (**A**; repeated-measures 2-way ANOVA, with Tukey’s post hoc test), distance traveled in the open field (**B**; 1-way ANOVA, with Tukey’s post hoc test), marbles buried (**C**; 1-way ANOVA, with Tukey’s post hoc test), nest building (**D**; repeated-measures 2-way ANOVA, with Tukey’s post hoc test), time spent floating during the forced swim task (**E**; Kruskal-Wallis test, with Dunn’s post hoc test), and audiogenic seizure susceptibility (**F**; Fisher’s exact test). *P < 0.05, **P < 0.01, ***P < 0.001, ****P < 0.0001.

We determined the functionality of transgenic UBE3A protein by testing its capacity to rescue behavioral deficits in AS model mice. We made use of a previously established behavioral test battery consisting of (sequentially) the rotarod, open field, marble burying, nest building, and forced swim tasks. Taken together, these tests consistently reveal motor dysfunction, deficits in species-typical innate behavior, and anxiety-like phenotypes in AS model mice (41, 42). If protein expressed from the *Ube3a^OE^* transgene is functionally competent, then it should prevent the manifestation of these phenotypes in double-transgenic mice resulting from a cross of the *Ube3a^OE^* line E and *Ube3a* (AS) mice (43). Indeed, AS/*Ube3a^+2^* double mutants were phenotypically indistinguishable from their WT counterparts (Figure 3, A–F), whereas AS mice exhibited obvious deficits in rotarod, nest building, marble burying, forced swim test, and audiogenic seizure susceptibility — a convincing replication of previous findings (41, 42). We found no statistically significant evidence of hypolocomotion in AS mice on the open field test (Figure 3B), though prior studies indicate that we were underpowered to detect this phenotype (42). Our capacity to resolve increased body weight was limited to female AS mice, in which the effect was also convincingly normalized in the AS/*Ube3a^+2^* group (Supplemental Figure 4, A–C). Separate studies of AS/*Ube3a^+1^* double mutants confirmed that a single *Ube3a^OE^* transgene copy was sufficient to restore UBE3A protein to WT levels in the brain (Supplemental Figure 5, A and B), fully rescuing increased female body weight, microcephaly, and impaired rotarod, marble burying, and nest building performance in AS littermate mice (Supplemental Figure 5, C–H). Collectively, these experiments demonstrate that the transgenic UBE3A protein expressed by *Ube3a^+1^* and *Ube3a^+2^* mice fully compensates for the loss of endogenous UBE3A, further establishing *Ube3a^OE^* lines as construct-valid models for exploring consequences of UBE3A overexpression relevant to the pathophysiology of Dup15q syndrome and broader neurodevelopmental contexts.

### Reciprocity of behavioral phenotypes in AS and Ube3a^OE^ mice.

**Figure 4:**
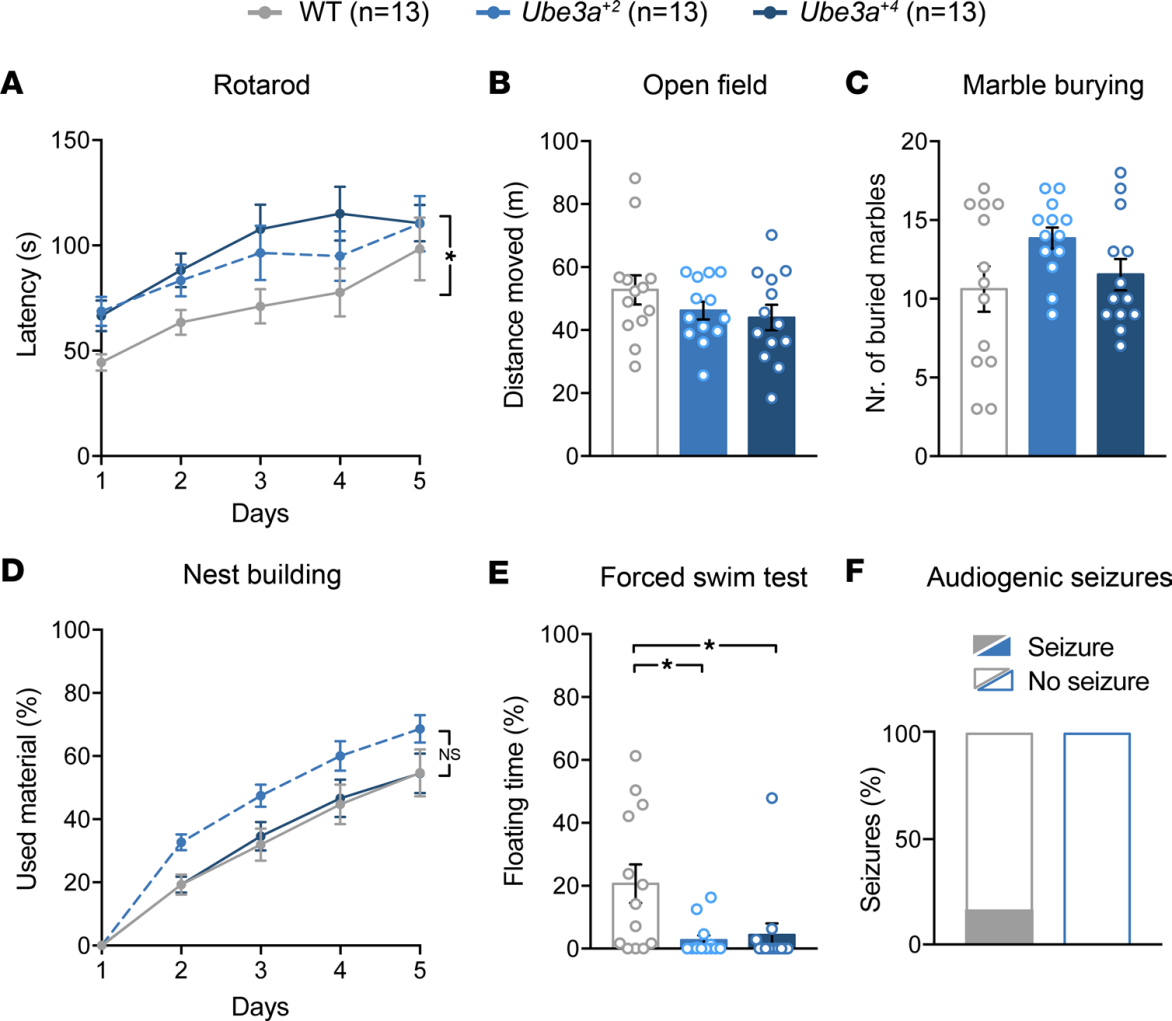
Transgenic UBE3A protein has limited behavioral consequences in *Ube3a^OE^* mice. (**A**–**F**) Mean ± SEM group performance of WT, *Ube3a^+2^*, and *Ube3a^+4^* mice for fall latency on the reversed rotarod task (**A**; repeated-measures 2-way ANOVA, with Tukey’s post hoc test), distance traveled in the open field (**B**; 1-way ANOVA, with Tukey’s post hoc test), marbles buried (**C**; 1-way ANOVA, with Tukey’s post hoc test), nest building (**D**; repeated-measures 2-way ANOVA, with Tukey’s post hoc test), time spent floating during the forced swim task (**E**; Kruskal-Wallis test, with Dunn’s post hoc test), and audiogenic seizure susceptibility (**F**; Fisher’s exact test). *P < 0.05.

AS mouse model phenotypes may reflect a requirement by specific neural circuitries (yet to be elucidated) for UBE3A expression levels to be maintained within an optimal range during their development. As such, we speculated that an overlapping group of tasks, including those that we used to assess the functionality of transgenic UBE3A protein in AS/*Ube3a^+2^* mice (Figure 3, A–F), might be impacted by both UBE3A loss of function and UBE3A overexpression. We were compelled to evaluate behavioral performance as a function of stepwise increases in *Ube3a* gene dosage, especially considering that 15q11.2–q13.1 copy number is a predictor of disease severity in Dup15q syndrome. Moreover, the absence of phenotypes in AS/*Ube3a^+2^* mice on these same tasks (Figure 3, A–F) — despite their having 1 extra *Ube3a* gene copy and expressing UBE3A protein in significant excess of WT throughout the brain (Supplemental Figure 4, D–G) — suggests a pathogenic threshold of 2 extra *Ube3a* copies, at least for this test battery. To this end, we crossed heterozygous *Ube3a^+2^* mice, generating WT, heterozygous *Ube3a^+2^*, and homozygous *Ube3a^+4^* offspring. This range of UBE3A overexpression was grossly well tolerated, as evidenced by a lack of body weight differences relative to WT in the *Ube3a^+2^* and *Ube3a^+4^* groups (Supplemental Figure 6, A–C). Behaviorally, we first subjected these cohorts to the accelerating rotarod task to assess motor learning and coordination capabilities. In contrast to AS mice, which consistently showed deficits on this task (Figure 3A) (42), we found a significantly increased fall latency in the *Ube3a^+4^* mice compared with WT controls (Figure 4A). Sequentially, we looked at performance in open field, marble burying, nest building, and audiogenic seizure susceptibility, finding no significant group differences (Figure 4, B–D and F). In the forced swim test, we again observed a phenotype opposite that of the AS model (Figure 3E), as floating time was significantly reduced for both *Ube3a^+2^* and *Ube3a^+4^* mice (Figure 4E); hence, this behavioral battery revealed a partial reciprocity of phenotypes in AS and *Ube3a^OE^* mice. The notable lack of phenotypic separation between *Ube3a^+2^* and *Ube3a^+4^* mice on these measures may be indicative of a ceiling effect for UBE3A overdosage. This may have a basis in the diminishing accumulation of UBE3A protein levels as *Ube3a* copies increase, as we observed both in our whole-brain analyses (Figure 1, D–G, and Supplemental Figure 2) and in this behavioral cohort across specific brain regions (Supplemental Figure 6, D–G).

### Analysis of Dup15q syndrome–relevant behaviors in Ube3a^OE^ mice.

Dup15q individuals generally present with moderate to profound forms of intellectual disability, ASD-like characteristics, and recurrent seizures that, in a minority of cases, result in sudden unexpected death in epilepsy (SUDEP) (11, 16, 19, 44–46). We therefore pursued further testing of *Ube3a^OE^* mice in behavioral domains relevant to these clinical symptoms of Dup15q syndrome.

**Figure 5:**
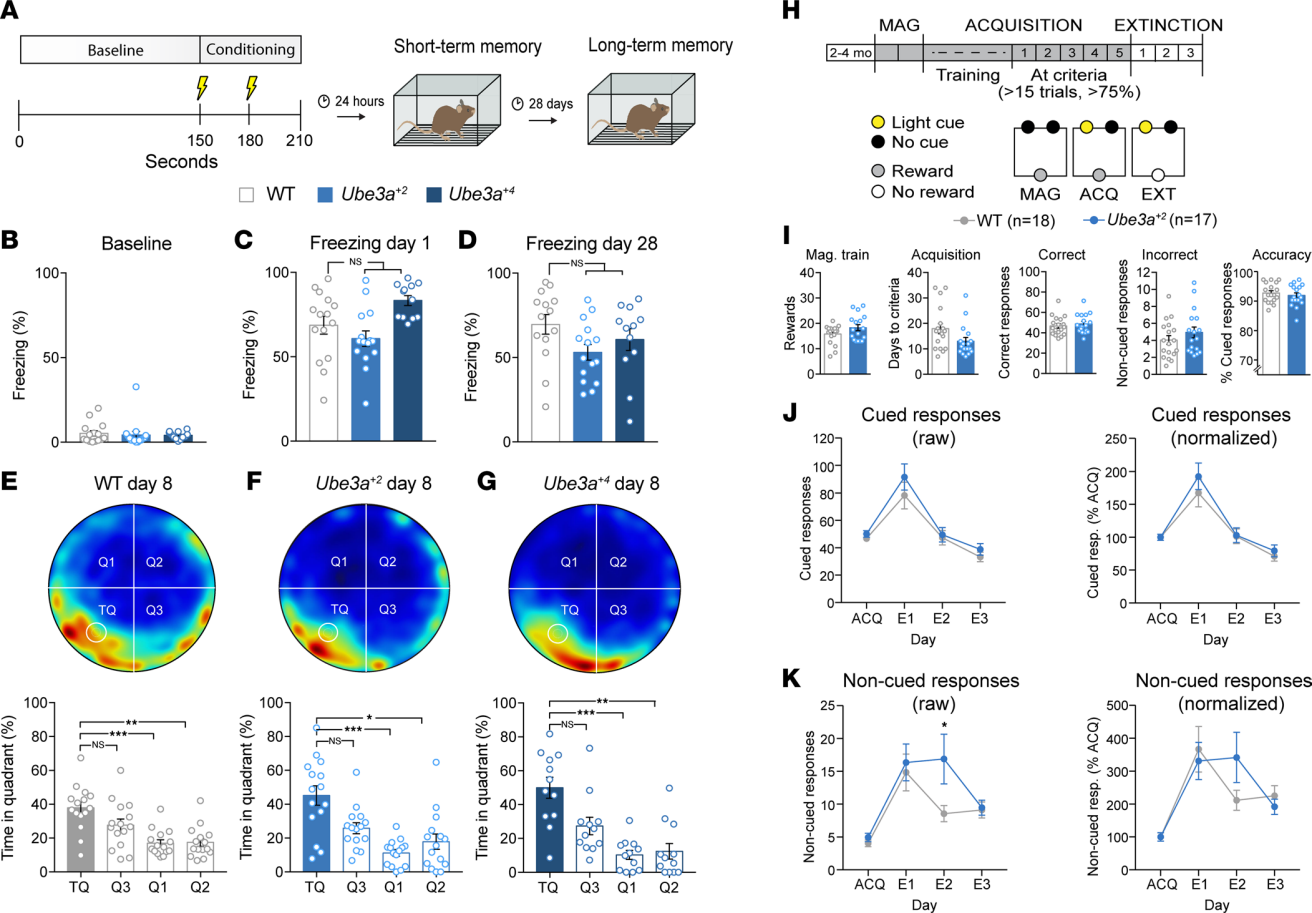
*Ube3a^OE^* mice show no deficits in cognitive performance. (**A**) Schematic of the fear conditioning paradigm. (**B**–**D**) Comparisons of context-specific freezing behavior among WT, *Ube3a^+2^*, and *Ube3a^+4^* mice. (**B**) Graph of baseline freezing expressed as percentage of total time. (**C** and **D**) Mean ± SEM freezing time 24 hours (**C**) and 28 days (**D**) after conditioning to ascertain short- and long-term fear-memory, respectively. One-way ANOVA and Tukey’s post hoc test. (**E**–**G**) Spatial memory acquisition as determined from the Morris water maze paradigm. Heatmap visualization and mean ± SEM graphing of average time spent by WT (**E**), *Ube3a^+2^* (**F**), and *Ube3a^+4^* (**G**) mice in maze quadrants Q1, Q2, and Q3 and the platform containing the target quadrant (TQ). Repeated-measures 1-way ANOVA, with Dunnett’s post hoc test. (**H**) Schematic of the experimental timeline for the magazine training (MAG), acquisition (ACQ), and extinction (EXT) tests (top) and of the operant conditioning chambers (bottom). Behavioral chambers contained a food magazine and 2 nose-poke apertures. Gray indicates the presence of food reward (MAG, ACQ); white indicates the absence of reward (EXT). Yellow indicates the presence of the light cue; black, its absence. (**I**) Left to right: Graphs of mean ± SEM rewarded nose pokes during MAG training, days to reach operant acquisition criteria, and raw correct and incorrect responses at ACQ criteria with computed response accuracy (proportion of correct responses to the cued aperture). Unpaired 2-tailed *t* test. (**J** and **K**) Graphs of mean ± SEM cued (**J**) and non-cued (**K**) responses over the last 5 days of ACQ and during each day of EXT. Left: Raw responses. Right: Normalized responses (to the group means of cued responses during the last 5 days of ACQ). Two-way ANOVA, Bonferroni’s post hoc. *P < 0.05, **P < 0.01, ***P < 0.001.

To evaluate cognitive function, we first subjected *Ube3a^OE^* mice to an associative learning and memory task. We implemented a classical fear conditioning paradigm based on determining a mouse’s capacity to associate an unanticipated, fear-provoking foot shock with a specific environmental context (learning), and to later recognize this context (memory) after both short and long intervals postconditioning (Figure 5A) (47, 48). Fear-associated freezing behavior among WT, *Ube3a^+2^*, and *Ube3a^+4^* mice was statistically similar, whether recorded at baseline or during short-term (24 hours) or long-term (28 days) contextual memory tests (Figure 5, B–D). These results show that associative learning and memory are unaffected by UBE3A overexpression.

We assessed spatial learning and memory using the Morris water maze task (49). Over the 7-day training phase, mice of all genotypes proved equally adept at using visual spatial cues outside the arena to locate the maze’s hidden escape platform (Supplemental Figure 7A). During probe trials (days 6 and 8), we removed the platform to gauge a mouse’s memory for its former location in the target quadrant (TQ). Heatmap-based visualization and quantification of probe trial swimming activity revealed an enrichment of TQ occupancy for WT, *Ube3a^+2^*, and *Ube3a^+4^* mice alike (Figure 5, E–G; and Supplemental Figure 7, D–F). The absolute number of TQ platform crosses was also similar across groups (Supplemental Figure 7, B and C). So, as with associative learning and memory, spatial memory acquisition appears to be unperturbed by UBE3A overdosage.

Hippocampal circuits principally subserve associative and spatial learning and memory (50, 51), and the long-term potentiation (LTP) of hippocampal synapses likely serves as a cellular substrate for these processes (52, 53). AS mice display severe impairments in hippocampal LTP (38, 54), thus establishing this measure as another potential cellular readout of altered UBE3A dosage. Accordingly, we measured LTP in hippocampal slices prepared from WT, *Ube3a^+2^*, and *Ube3a^+4^* mice (Supplemental Figure 7G), further extending our functional studies. Here too, mice of both *Ube3a^OE^* groups were utterly indistinguishable from WT controls.

To further investigate potential effects of UBE3A overexpression on cognitive function, we compared the performance of WT and *Ube3a^+2^* mice on a task that was previously used to demonstrate enhanced operant extinction in both Angelman and fragile X syndrome model mice (55, 56). Three stages of testing — (a) magazine training, (b) operant acquisition, and (c) operant extinction — ensued, with mice performing subsequent to food restriction, thus motivated to receive food pellet rewards (Figure 5H). WT and *Ube3a^+2^* mice exhibited statistically equivalent nose-poke responding for food rewards during magazine training (Figure 5I), suggesting no difference in motivation or reward salience between groups. During operant acquisition, mice learned to nose-poke the illuminated aperture for reward (cued response), and we tested them daily until they achieved predefined criteria: >15 trials and >75% accuracy for 5 consecutive days (Figure 5H). Notably, *Ube3a^+2^* mice did not differ from their WT counterparts in either rate of operant acquisition or response accuracy at criteria (Figure 5I). Following acquisition, mice underwent 3 days of extinction testing in the absence of food reward. For raw or normalized data, both genotypic groups showed similar extinction learning dynamics, with high rates of cued responding on day 1 (E1) that rapidly tapered off over the subsequent 2 days of testing (E2–E3) (Figure 5J). *Ube3a^+2^* mice did make significantly more non-cued responses than WT on E2, but this effect was statistically nonsignificant when responding during extinction was normalized to the average responding rate during the acquisition phase (Figure 5K). Considering that *Ube3a^+1^* mice were similarly indistinguishable from WT with respect to operant acquisition and extinction learning (Supplemental Figure 8), the evidence supports that the underlying neural processes are impervious to UBE3A overexpression to the degree tested here. This adds to our overall conclusion that cognitive function in *Ube3a^OE^* mice is largely intact.

**Figure 6:**
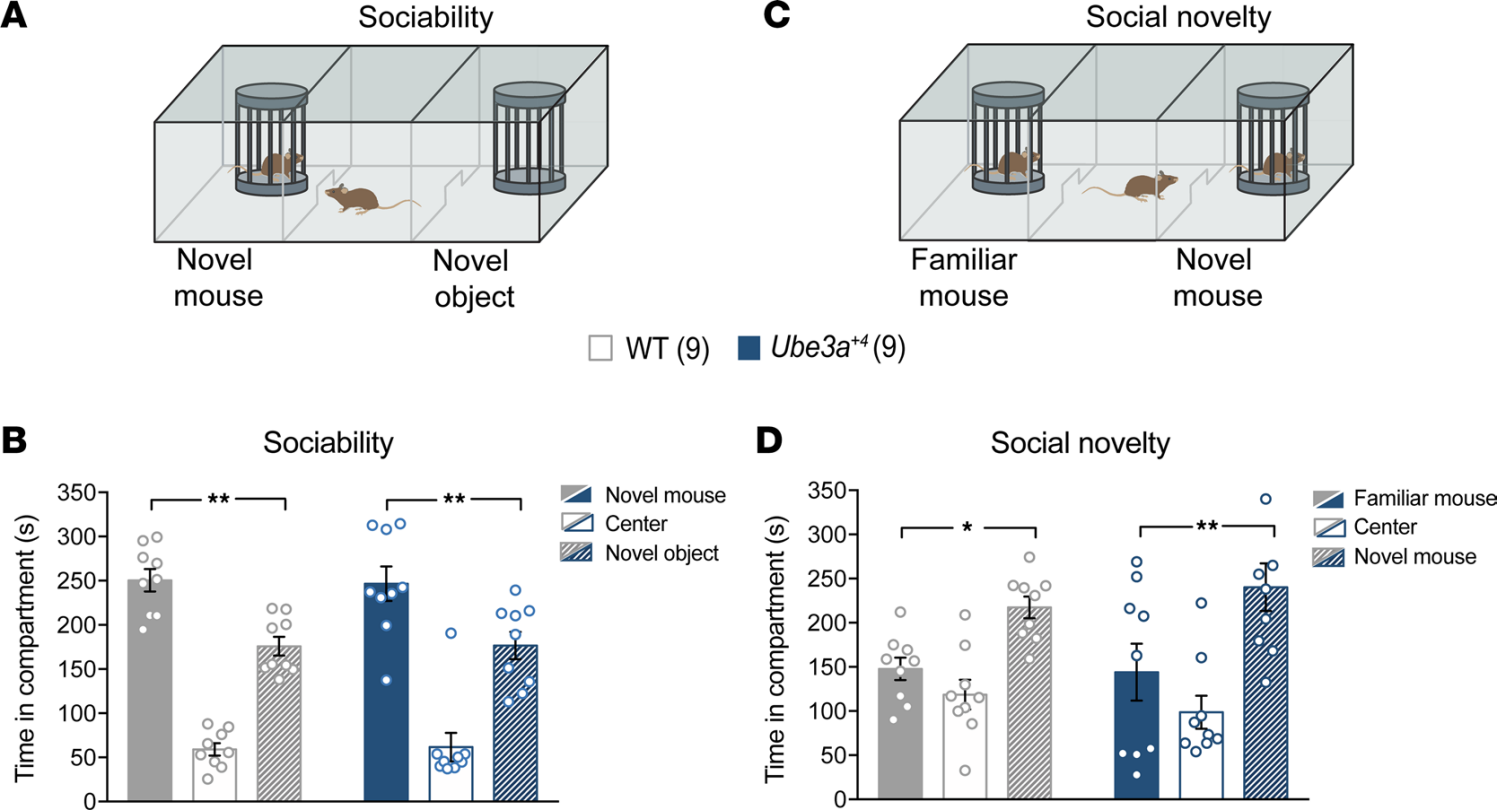
*Ube3a^OE^* mice display normal social behavior in a 3-chamber sociability test. (**A**) Schematic of the 3-chamber paradigm used to assess sociability in WT versus *Ube3a^+4^* mice. (**B**) Mean ± SEM time spent in compartments containing the novel mouse (filled bars), the novel object (striped bars), or center (open bars). Two-way ANOVA, Holm-Šidák post hoc test. (**C**) Schematic of the 3-chamber paradigm used to assess social novelty preference. (**D**) Mean ± SEM time spent in compartments containing the familiar mouse (filled bars), the novel mouse (striped bars), or center (open bars). Two-way ANOVA, Holm-Šidák post hoc test. *P < 0.05, **P < 0.01.

To potentially capture phenotypes relevant to social deficits in ASD, we exposed *Ube3a^+4^* mice to the 3-chamber social interaction test (57–59). In tests of sociability, this paradigm pits a mouse’s preference for investigating an unfamiliar conspecific against its tendency to explore a novel object (Figure 6A). Inbred mice of various strains have been shown to spend more time investigating the novel mouse than the novel object, thereby establishing the face validity of this task (60). As an extension of the sociability test, social novelty preference can be assessed. This is achieved by replacement of the novel object with a second unfamiliar conspecific and analysis of whether investigation disproportionately shifts toward the newly introduced mouse (Figure 6C). We tested sociability and social novelty preference in WT versus *Ube3a^+4^* mice. In the sociability task, both groups demonstrated a significant tendency to occupy the novel-mouse-containing compartment (Figure 6B) but were statistically comparable in terms of this sociability preference. WT and *Ube3a^+4^* mice also proved to similarly favor investigations of unfamiliar mice in the course of social novelty testing (Figure 6, C and D). Hence, these experiments failed to reveal any UBE3A dose–driven effect on social behavior.

**Figure 7:**
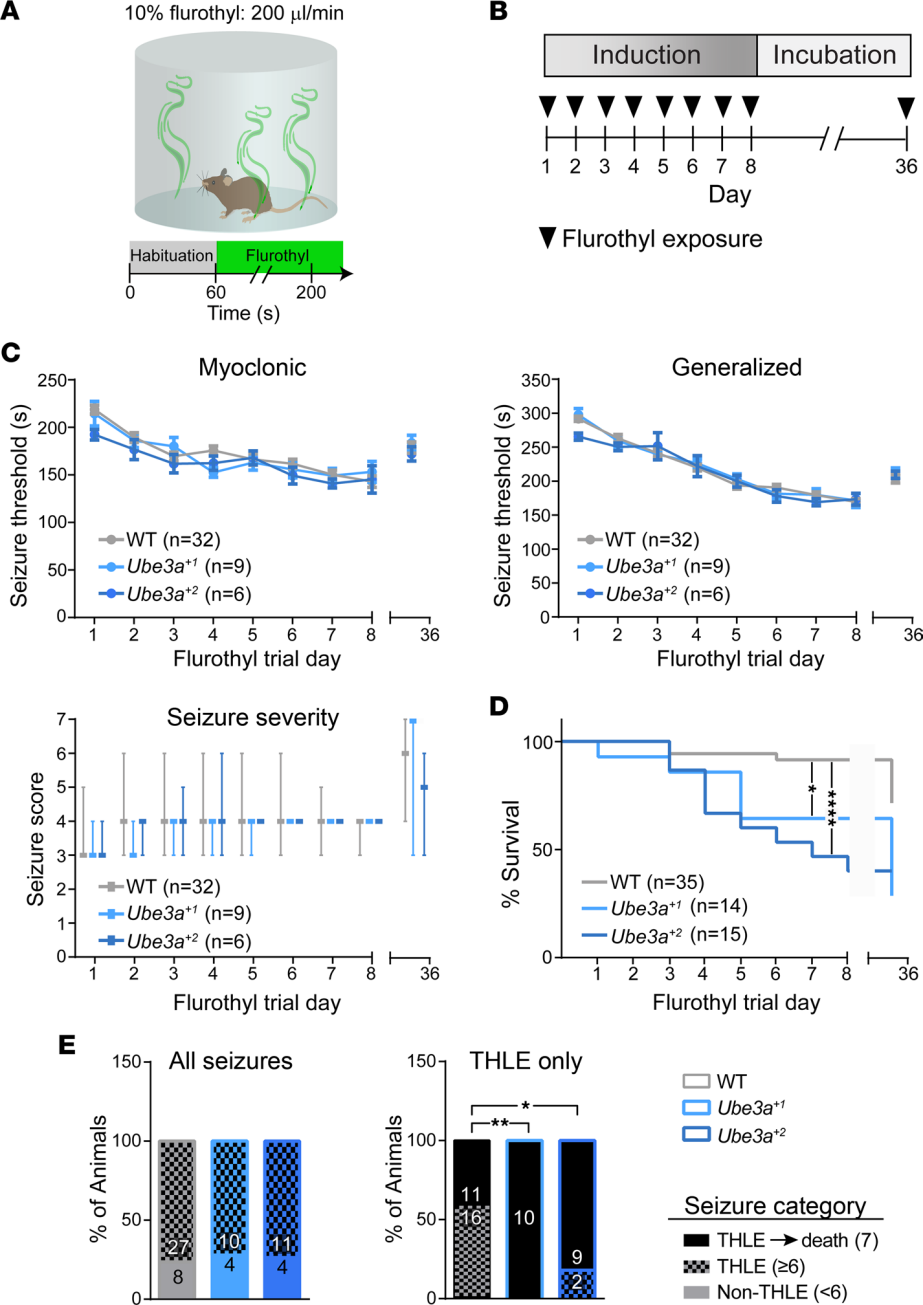
*Ube3a^OE^* mice exhibit enhanced susceptibility to seizure-associated death during flurothyl kindling. (**A**) Schematic of flurothyl-induced seizure protocol. (**B**) Schematic of experimental paradigm for 8-day flurothyl seizure kindling and rechallenge. (**C**) Graphs of mean ± SEM latencies to myoclonic (top left) and generalized (top right) seizure depicting changes in seizure threshold throughout flurothyl kindling and rechallenge, analyzed by 2-way repeated-measures ANOVA, Tukey’s post hoc; and graph of median and range for generalized seizure severity based on a modified Racine scale (bottom), analyzed by mixed-effects generalized linear modeling (see Supplemental Figure 10 for details). Data represent mice surviving all 8 days of flurothyl kindling. (**D** and **E**) Group survival (**D**) and proportionality of seizure severity (**E**) for all tested mice. The left panel in **E** depicts the proportion of mice experiencing at least 1 seizure with tonic hind limb extension (THLE; score of 6 or 7 on modified Racine scale) versus the proportion not experiencing any (Non-THLE, score <6); the right panel depicts the proportion of mice in which THLE seizures progressed to death (score of 7). Survival curves were compared with the log-rank (Mantel-Cox) test. The proportionality of seizure severity was analyzed using χ^2^ statistics. *P < 0.05, **P < 0.01, ****P < 0.0001.

*Ube3a^+2^* mice showed no evidence of a lowered audiogenic seizure threshold (Figure 4F). We wondered whether this finding might generalize to other seizure induction paradigms, and furthermore, whether UBE3A overexpression would not exacerbate susceptibility to epilepsy following seizure kindling. Therefore, we subjected integrated cohorts of *Ube3a^+1^*, *Ube3a^+2^*, and WT littermate mice from line E to the repeated flurothyl seizure model (61). Flurothyl seizure induction once daily for 8 days, with rechallenge at day 36, allows for the assessment of both ictogenic (day 1) and epileptogenic (days 2–8 and day 36) potential in mice (Figure 7, A and B) (62, 63). Compared with WT, neither *Ube3a^+1^* nor *Ube3a^+2^* mice exhibited a significantly lower induction threshold for either myoclonic or generalized seizures on day 1 (Figure 7C). This result corroborated our audiogenic seizure data (Figure 4F), further supporting that UBE3A overexpression alone is insufficient to enhance baseline seizure susceptibility in naive mice. Over the next 7 days, both *Ube3a^OE^* groups kindled at a similar rate to WT, and all groups displayed equivalent seizure thresholds 28 days later during rechallenge (Figure 7C). This contrasted sharply with what we had previously reported for AS mice (64) and indicated that UBE3A overexpression per se is not a pro-epileptogenic factor.

*Ube3a^OE^* mice had flurothyl-induced seizures no more readily than WT, but they were far more likely to die from them: approximately 35% of *Ube3a^+1^* mice and more than 50% of *Ube3a*^+2^ mice died during the 8-day induction period, whereas WT mice seldom succumbed (Figure 7D). We also observed increased seizure mortality in line C *Ube3a^+1^* and *Ube3a^+2^* mice compared with WT controls despite modest (*Ube3a^+1^*) or no (*Ube3a^+2^*) differences in baseline flurothyl seizure susceptibility or rates of flurothyl kindling (Supplemental Figure 9). This seizure-associated death phenotype did not seem to be a product of increased seizure severity. Seizure severity scores were similar across groups (Figure 7C and Supplemental Figure 10), and equivalent proportions of WT, *Ube3a^+1^*, and *Ube3a^+2^* mice experienced the most extreme class of seizures involving tonic hind limb extension (THLE), rating ≥6 on a modified Racine scale. However, in contrast to WT mice, when *Ube3a^OE^* mice had THLE seizures, they rarely survived them (Figure 7E). This striking phenotype may have implications for SUDEP as sometimes occurs in individuals with Dup15q syndrome (46).

### Transcriptomic effects of UBE3A overexpression.

**Figure 8:**
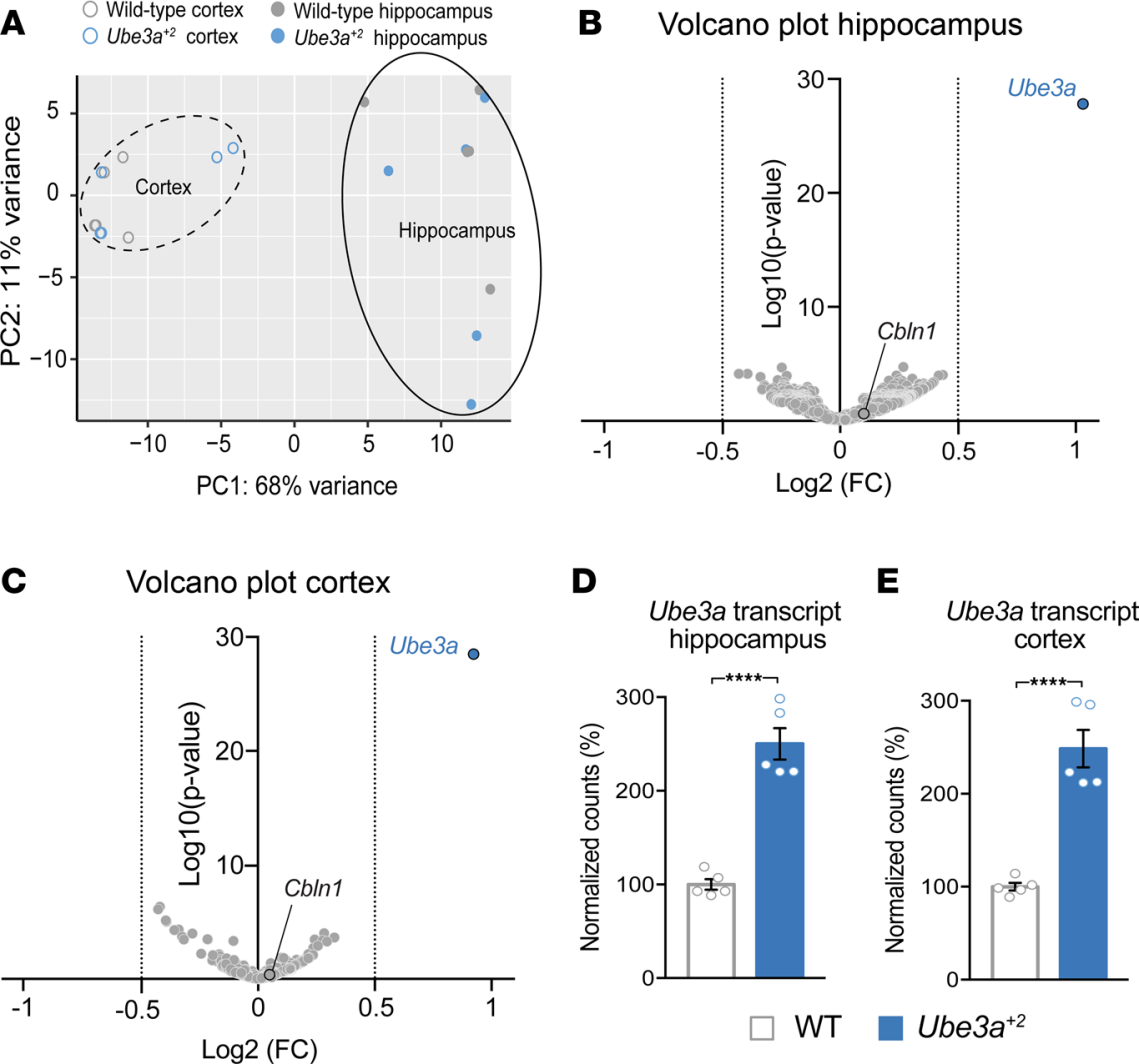
RNA-Seq reveals no significant transcriptional changes due to UBE3A overexpression. (**A**) Principal component analysis plot demonstrating the sample clustering of cortical and hippocampal tissue taken from WT and *Ube3a^+2^* mice at P7. Open circles represent cortex and filled circles represent hippocampus. Clustering of cortical and hippocampal samples is indicated by dashed and solid outlines, respectively. (**B** and **C**) Volcano plots of the differential gene expression analysis performed on samples of hippocampal (**B**) and cortical (**C**) origin of the 1,000 genes with the lowest *P* values. Log_2_ fold change is plotted on the *x* axis versus log_10_ *P* value on the *y* axis. Dashed lines indicate the –0.5 and 0.5 log_2_ fold change borders. The *Ube3a* data points are indicated in blue, while the previously identified RNA target, *Cbln1* (22), is outlined in black. (**D** and **E**) Normalized *Ube3a* transcript counts in hippocampus (**D**) and cortex (**E**). Data are presented as percentages, with WT transcript counts set at 100%. The normalized counts were compared using an unpaired 2-tailed *t* test. ****P < 0.0001.

Given the evidence that UBE3A coregulates several transcription factors (32–34), and the potential for secondary and tertiary transcriptomic effects resulting from UBE3A-mediated changes in protein homeostasis (30), UBE3A overdosage may possibly lead to the differential expression of numerous transcripts. In support of this hypothesis, multiple studies have linked UBE3A overdosage to transcriptomic changes, some potentially with disease-causing consequences (e.g., Cbln1) (22, 35). We investigated transcriptional changes in our *Ube3a^+2^* model, performing transcriptome-wide analysis of the developing hippocampus and cortex via RNA-Seq. In line with our behavioral observations, but contrary to previous literature, our experiments revealed modest consequences of UBE3A overexpression. Principal component analysis of the individual samples showed profound clustering according to anatomical region but not genotype (Figure 8A). Remarkably, tissue-specific differential gene expression analysis comparing WT and *Ube3a^+2^* mice indicated *Ube3a* as the only transcript to be significantly deregulated (adjusted *P* value < 0.05; log_2_ fold change > 0.5 or < –0.5), in both hippocampus (Figure 8, B and D) and cortex (Figure 8, C and E) (see also Supplemental Data 1). Together, these findings depict transcriptional regulation as largely intact in the aftermath of significant UBE3A overexpression.

## Discussion

Dup15q syndrome is most prevalent and of greatest clinical severity in individuals with 15q11.2–q13.1 duplications of a maternal (as opposed to a paternal) origin, making it likely that epigenetic factors and monoallelically expressed 15q11.2–q13.1 genes are principal pathophysiological drivers of this disorder (26). As *UBE3A* is the only gene in this chromosomal region expressed solely from the maternal gene copy, we aimed here to selectively elucidate the pathological consequences of its overexpression. We based our efforts on *Ube3a^OE^* model mice harboring exactly 1, 2, or 4 extra copies of the entire *Ube3a* gene, precisely reflecting levels of UBE3A overdosage associated with 15q duplication (5), triplication (3), and hexasomy (65–68), respectively. Following rigorous experiments to confirm transgenic UBE3A expression in the model, we were intrigued to discover that *Ube3a^+4^* mice, which harbor 5 transcriptionally active *Ube3a* copies in neurons, express just over 300% of the UBE3A protein level observed in WT brain; 500% of WT brain UBE3A content would be expected in *Ube3a^OE^* model mice if UBE3A protein increases were perfectly additive. A plausible explanation for this observation is that UBE3A, itself an E3 ligase capable of self-ubiquitination, autoregulates its own levels through ubiquitin proteasome–mediated degradation (69). At higher levels of expression, UBE3A might interact with itself with greater frequency, increasing the likelihood of self-degradation. Although this is an attractive hypothesis, we found *Ube3a* mRNA levels in *Ube3a^OE^* mice to be similarly limited in response to escalating *Ube3a* gene dosage. This may be indicative of a transcriptional, rather than a posttranslational, feedback mechanism — one in which UBE3A negatively regulates its own expression.

Extensive molecular, electrophysiological, and behavioral testing in the *Ube3a^OE^* model has led us to the overarching conclusion that UBE3A overexpression is generally well tolerated by the developing nervous system; by and large, our experiments failed to detect phenotypic deficits in *Ube3a^OE^* mice relative to WT controls. *Ube3a^OE^* mice actually outperformed their WT counterparts on the rotarod and showed a reduced tendency to float during the forced swim task. This is opposite of AS mice, which exhibited poor rotarod performance and increased floating in the forced swim task, suggesting that in at least some cases, loss of UBE3A expression and UBE3A overdosage mediate reciprocal phenotypic outcomes. It is also noteworthy that increased UBE3A dosage rendered *Ube3a^OE^* mice challenged with a course of flurothyl seizure kindling more susceptible to seizure-associated death, a phenotype reminiscent of SUDEP in Dup15q individuals (11, 16, 19, 44–46). Because enhanced seizure-associated death proved to replicate in 2 independent *Ube3a^OE^* lines, it is most likely a genuine consequence of UBE3A overexpression, not a spurious effect of transgene integration. Further flurothyl kindling experiments testing the full range of *Ube3a* gene overdosage possible in *Ube3a^OE^* mice (i.e., up to *Ube3a^+6^*) will be required to determine whether this is truly a *Ube3a* dose–sensitive phenotype.

Our findings contradict those from alternative mouse models of UBE3A overexpression, in which elevated UBE3A levels were associated with numerous behavioral abnormalities ranging from learning deficits, increased anxiety-like behavior, and reduced seizure thresholds (21) to core ASD features including impaired sociability and repetitive behavior (20, 22). This incongruity likely stems from differences in model design.

Initially, Anderson and colleagues produced mice overexpressing UBE3A with a C-terminal FLAG tag (20). Additional models were produced by this same laboratory in follow-up studies — some overexpressing UBE3A with tandem FLAG tags and nuclear localization signals at the C-terminus, others overexpressing untagged, presumably functional UBE3A protein (22). Because fusions made to the UBE3A C-terminus eliminate the catalytic activity of the protein (70, 71), the collective body of work based on these mice is difficult to interpret. Although it was argued that the inactivity of tagged UBE3A protein could be overcome by its being incorporated into a hetero-multimer of endogenous and transgenic UBE3A (22, 72), evidence of such a mechanism is lacking. Moreover, one could with similar ease envision that multimerization of active and inactive UBE3A molecules produces a significant dominant-negative effect, diminishing overall UBE3A enzymatic function and tending toward a scenario reminiscent of Angelman syndrome. Each UBE3A overexpression model generated by Anderson and colleagues exhibited strikingly similar transcriptomic perturbations as well as pronounced deficits in social behavior (22), despite the aforementioned differences in the laboratory’s transgenic UBE3A designs and the as-yet-unknown influences of variable, overly excessive *Ube3a* copy number (4–9 copies) and (potentially) unique off-target effects of transgenic insertion — each likely to be exacerbated by breeding to homozygosity as was the case in these studies. If valid, an essential implication of these findings is that UBE3A E3 ligase activity (a presumed but unverified feature of the untagged Anderson model) is dispensable for the observed pathophysiological effects. This possibility, and what (if any) relevance it may have to pathogenic mechanisms in Dup15q syndrome, remain to be directly tested. Notably, enzymatically competent UBE3A is essential to prevent Angelman syndrome pathogenesis (30, 31, 73).

For their part, Copping and colleagues (21) ingeniously generated a mouse model that features isoform- and neuron type–restricted UBE3A overexpression in the brain, harnessing *CamK2a*-driven tetracycline transactivation to induce *Ube3a* isoform 2 specifically in forebrain excitatory neurons. While useful for identifying UBE3A isoform–specific functions, this approach has its own limitations with respect to construct validity for UBE3A overexpression in Dup15q syndrome. UBE3A isoform 2 is exclusively localized to the cytoplasm (74, 75), and recent work examining Angelman syndrome–associated missense mutations suggests that cytoplasmic localization of UBE3A at the expense of its nuclear targeting is a predictor of pathogenicity, irrespective of catalytic function (73). N-terminal FLAG tagging of UBE3A isoform 2, another feature of this model, may have further, untold consequences for the intracellular function of this transgenic protein. Additionally, at the circuit level, imbalanced UBE3A expression among excitatory and inhibitory forebrain neurons may lead to an atypical exacerbation of seizure phenotypes, as has been demonstrated in conditional *Ube3a* deletion experiments (76).

By comparison, the transgenic UBE3A protein in our *Ube3a^OE^* model mice is native, untagged, and expressed according to endogenous spatiotemporal patterns of expression; it is also fully functional, as demonstrated by its capacity to fully rescue behavioral phenotypes due to the loss of endogenous UBE3A in AS model mice, evidence that has yet to be provided for any of the other transgenic approaches. This is presumably the modeling scenario that most closely reflects UBE3A overexpression per se in Dup15q syndrome.

Mice harboring a chromosome 7 interstitial duplication (syntenic to the human 15q11–13 duplication) have enabled studies of UBE3A overexpression in concert with the overexpression of several other genes in the 15q11.2–q13.1 region (36). By all appearances, this model, the product of an impressive feat of chromosome engineering, provides excellent construct validity for Dup15q syndrome. Nevertheless, its phenotypic profile is somewhat perplexing with respect to parent-of-origin inheritance of the duplication. Mice with paternal inheritance (patDup) express a subset of autism-related phenotypes that seem to depend on the overexpression of paternally expressed driver genes (77). In contrast, mice with maternal inheritance (matDup) are largely normal despite confirmed overexpression of *Ube3a* and nearby nonimprinted genes (36). This clashes with the clinical reality of Dup15q syndrome, in which maternally inherited duplications are generally most phenotypically penetrant (11, 15, 26). What could explain this discrepancy? Simply, there may exist a higher phenotypic threshold in mice (relative to humans) for the maternal duplication of homologous 15q11.2–q13.1 genes. Idic(15) levels of overexpression may be required to significantly impact neurodevelopmental trajectories. This notion had motivated our choice to explore the consequences of incremented genetic *Ube3a* overexpression, using *Ube3a^+2^* and *Ube3a^+4^* mice. That we observed a lack of cellular and behavioral phenotypes despite such marked increases in *Ube3a* gene copy number speaks to a relatively robust neurodevelopmental tolerance of UBE3A overdosage in mice, at least in the absence of concomitant overexpression of nonimprinted 15q11.2–q13.1 gene homologs. Succinctly, given both the available clinical data detailing relatively mild neuropsychiatric outcomes in cases of *UBE3A* microduplication (27), and our findings in the *Ube3a^OE^* models, UBE3A overexpression appears to be necessary, but not sufficient, to drive the pathophysiological mechanisms that underlie Dup15q syndrome.

Of the possible 15q11.2–q13.1 genes that may co-contribute to the manifestation of Dup15q syndrome, *HERC2* is an especially promising candidate. HERC2 protein, itself a HECT E3 ubiquitin ligase, has been shown in vitro to physically interact with — and modulate the catalytic activity of — UBE3A (78). A destabilizing *HERC2* missense mutation was reported to be associated with Angelman-like features (79, 80), leading Harlalka and colleagues to postulate that lower HERC2 levels translate to insufficient UBE3A activity in the affected individuals. The implication of UBE3A-HERC2 codependency for Dup15q syndrome is that a concomitant increase of HERC2 levels is required to fully activate pools of overexpressed UBE3A, which in turn produce pathogenic effects. Overexpression of the 3 γ-aminobutyric acid type A (GABA_A_) receptor genes in the 15q11.2–q13.1 region has been strongly linked to excessive β oscillations in Dup15q individuals. This EEG phenotype, which mimics the effects of benzodiazepine treatment, is equally penetrant in maternal and paternal Dup15q and thus occurs independently of UBE3A overdosage (81). Yet, enhanced beta or other manifestations of increased GABAergic tone could conceivably add to, or compound, UBE3A-driven Dup15q pathophysiologies. The impact could be substantial and warrants further investigation. Combinatorial overexpression of *Ube3a* and other 15q11.2–q13.1 genes in mouse models represents a plausible approach to elucidating Dup15q syndrome disease mechanisms but one that will be laborious, time-consuming, and costly. Therefore, it will be essential to pursue in parallel complementary studies of cellular phenotypic rescue following combinatorial normalization of 15q11.2–q13.1 gene expression levels in neurons differentiated from Dup15q patient-derived induced pluripotent stem cells (82).

There is evidence that maternal duplications shift the epigenetic balance of the 15q11.2–q13.1 region, thereby influencing the expression of genes therein in a manner not predicted by copy number. For instance, 15q11.2–q13.1 duplications encompass the *GABRB3* gene, yet increased GABRB3 protein levels were not evident from postmortem brain analyses. More strikingly, *SNRPN* RNA expression levels were observed to be lower in Dup15q cortical samples than in neurotypical controls and individuals with ASD (83, 84). Considering that *SNRPN* is exclusively expressed from the paternal allele (85, 86), it was highly unexpected that its expression would be affected by a duplication of maternal origin. The *SNRPN* gene is part of the critical Prader-Willi syndrome (PWS) region, of which deletion leads to PWS, a neurodevelopmental disorder characterized by hypotonia, feeding difficulties, motor delay, and cognitive impairments (13). Additionally, SNRPN knockdown in neurons is reported to affect neurite outgrowth, neuron migration, and spine distribution (87). Further studies will be required to determine whether decreased SNRPN expression is a pathogenic factor in Dup15q syndrome, and if so, how it might be brought about by the overexpression of *UBE3A* and possibly other 15q11.2–q13.1 genes.

Conclusively, our study calls for a refined perspective on contributions of UBE3A overexpression to the etiology of Dup15q syndrome. We advocate for an adapted viewpoint, one that continues to favor *UBE3A* as a critical driver of pathophysiology but emphasizes its cooperativity with other supernumerary 15q11.2–q13.1 genes to this effect. This viewpoint lends itself to a favorable therapeutic outlook: with cooperating pathogenic players, each may individually offer the opportunity for outsized relief of Dup15q syndrome symptomology upon genetic normalization.

## Methods

A detailed description of the methods can be found in Supplemental Methods. All RNA-Seq data were deposited in the NCBI’s Gene Expression Omnibus database (GEO GSE205128; https://www.ncbi.nlm.nih.gov/geo/query/acc.cgi?acc=GSE205128). Full, uncut images of protein blots are included in the published online supplemental material.

### Statistics.

All statistical analyses were done using GraphPad Prism software (v7.0 for Macintosh, GraphPad Software Inc., RRID:SCR_002798), except for models of changing seizure severity in the course of flurothyl kindling, which were estimated using GLIMMIX in SAS (v9.4, SAS Institute Inc., RRID:SCR_008567). All data are presented as mean ± SEM unless specified otherwise; *P* less than 0.05 was considered significant. Supplemental Data 2 contains detailed information on all test statistics and *P* values.

### Study approval.

All experiments were performed in strict compliance with animal protocols approved by the Institutional Animal Care and Use Committee of the University of North Carolina at Chapel Hill and in accordance with European Commission Council Directive 2010/63/EU (CCD approval AVD101002016791).

## Author contributions

MCJ designed and validated the mouse model and performed Western blotting, quantitative PCR, immunofluorescence, and flurothyl kindling experiments. BNW and PFL performed and analyzed flurothyl kindling experiments. AMP, SB, and DDH performed behavior experiments and corresponding Western blotting experiments. MSS and NSJ performed operant extinction experiments. CRD performed and analyzed quantitative PCR experiments. MSF performed and analyzed Western blotting experiments. PJC performed statistical analysis of flurothyl-induced seizure severity. AMP and WFJVI performed and analyzed the RNA-Seq experiment. MAJ performed the LTP experiments. BDP and YE supervised the project. AMP, MCJ, BDP, and YE wrote the manuscript. All authors reviewed the manuscript. The order of co–first authors and of co–senior authors was determined based on their overall contribution to the study.

## Supplementary Material

Supplemental data

Supplemental data set 1

Supplemental data set 2

Supplemental data set 3

## Version 1 09/22/2022

Electronic publication
