# Supplementary material for: Molecular and behavioral consequences of Ube3a gene overdosage in mice
Source: JCI Insight. 2022 Sep 22;7(18):e158953. doi: 10.1172/jci.insight.158953 (PMC9675564; doi:10.1172/jci.insight.158953)
Supplement: Supplemental data [file jciinsight-7-158953-s066.pdf]

Molecular and behavioral consequences of *Ube3a* gene overdosage in mice

Supplemental figures

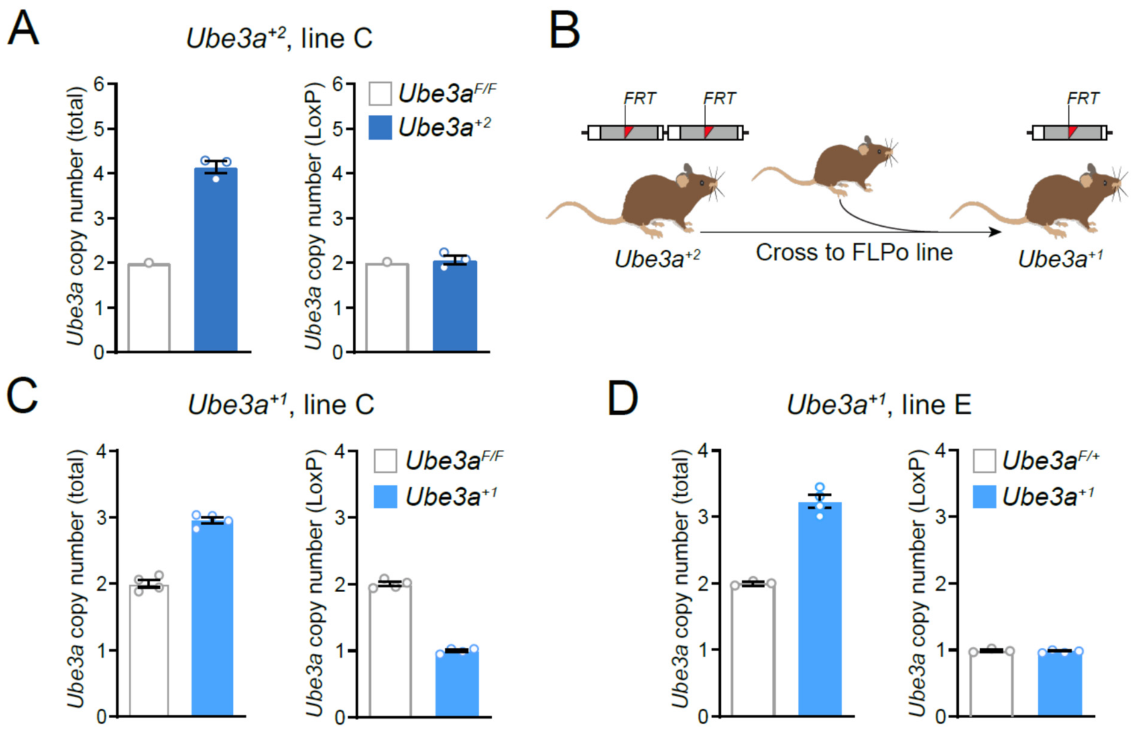

Figure S1. Generation and genomic characterization of *Ube3a*$^{+1}$ mice. (**A**) Droplet digital PCR (ddPCR) analysis of total and transgenic *Ube3a* genomic copy number in line C *Ube3a*$^{+2}$ overexpression mice. ddPCR primers and probes specific to the intron 3/exon 3 boundary of endogenous *Ube3a* were used to assess total (i.e., endogenous + transgenic) *Ube3a* copy number (left panel); ddPCR primers and probes recognizing loxP sequences enabled specific detection of transgenic *Ube3a* copies (right panel). (**B**) Schematic of strategy used to obtain *Ube3a*$^{+1}$ sublines from *Ube3a*$^{+2}$ mice. The FLPo allele was bred out of *Ube3a*$^{+1}$ mice prior to genomic characterization. (**C-D**) ddPCR analysis of total and transgenic *Ube3a* genomic copy number in line C *Ube3a*$^{+1}$ (C) and line E *Ube3a*$^{+1}$ overexpression mice (D). Heterozygous (*Ube3a*$^{F/+}$) and homozygous (*Ube3a*$^{F/F}$) floxed *Ube3a* knock-in mice served as controls for ddPCR experiments.

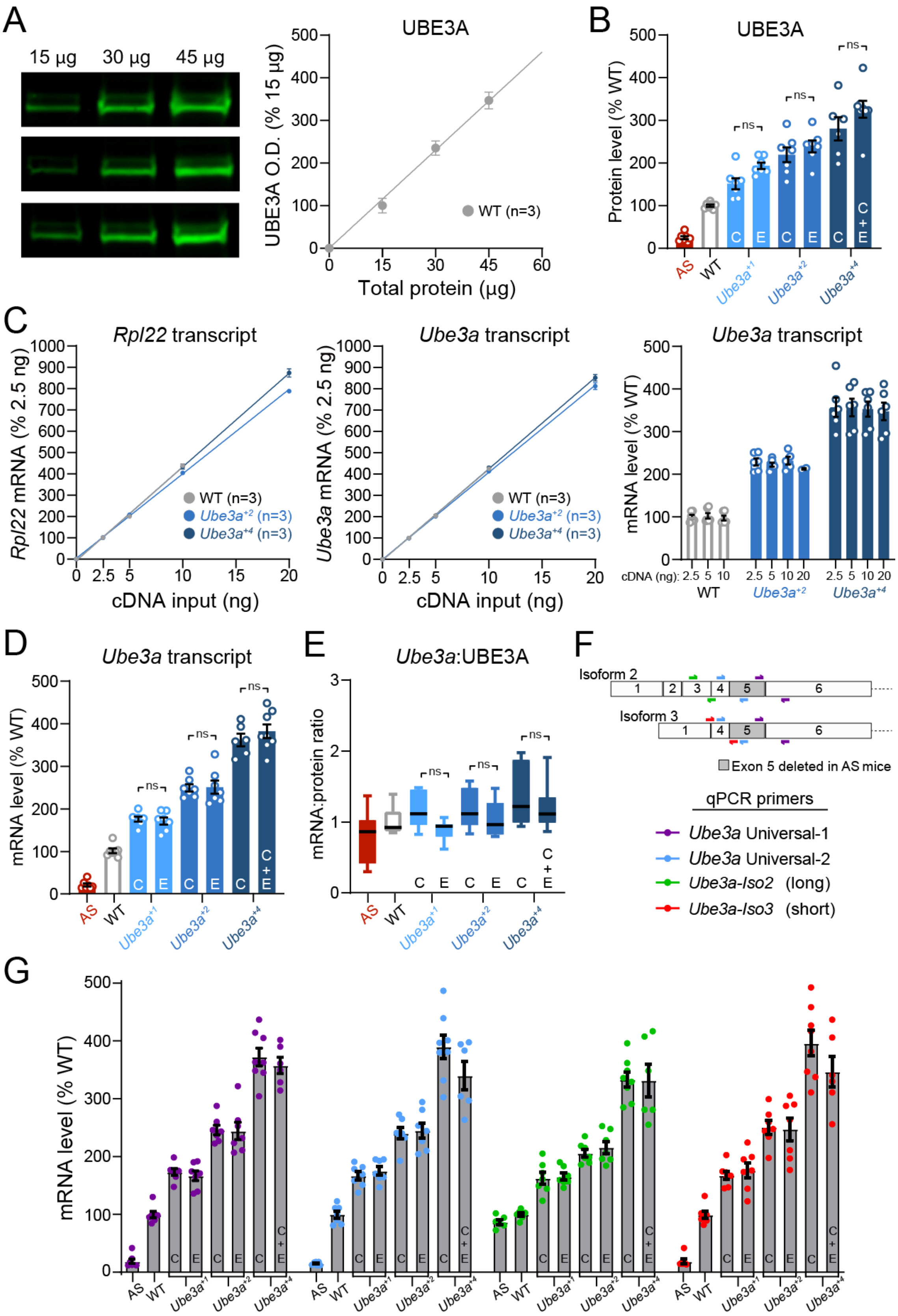
A
15 µg
30 µg
45 µg
UBE3A
UBE3A O.D. (% 15 µg)
Total protein (µg)
WT (n=3)
B
UBE3A
Protein level (% WT)
ns
AS
WT
Ube3a+1
Ube3a+2
Ube3a+4
C
E
C + E
C
Rpl22 transcript
Rpl22 mRNA (% 2.5 ng)
cDNA input (ng)
WT (n=3)
Ube3a+2 (n=3)
Ube3a+4 (n=3)
Ube3a transcript
Ube3a mRNA (% 2.5 ng)
mRNA level (% WT)
cDNA (ng):
D
Ube3a transcript
mRNA level (% WT)
E
Ube3a:UBE3A
mRNA:protein ratio
F
Isoform 2
Isoform 3
Exon 5 deleted in AS mice
qPCR primers
Ube3a Universal-1
Ube3a Universal-2
Ube3a-Iso2 (long)
Ube3a-Iso3 (short)
G
mRNA level (% WT)

Fig. S2. Dynamic range of UBE3A Western blot and *Ube3a* ddPCR assays, and *Ube3a*^{OE} line-specific expression of UBE3A protein and *Ube3a* transcript. (**A**) Left: replicate Western blot (WB) experiments depicting immunofluorescent visualization of UBE3A in WT whole-brain lysates loaded with increasing levels of total protein. Right: relative quantification of blots at left demonstrating reliable detection of up to at least 2-fold increases in UBE3A protein content. WB experiments in main Fig. 1 were loaded at 15 µg of total protein. (**B**) Mean ± SEM UBE3A WB immunofluorescence intensities as reported in main Fig. 1, broken out by specific *Ube3a*^{OE} lines: line C *Ube3a*^{OE}—*Ube3a*$^{+1}$ (N=7), *Ube3a*$^{+2}$ (N=7), and *Ube3a*$^{+4}$ (N=6)—and line E *Ube3a*^{OE}—*Ube3a*$^{+1}$ (N=7), *Ube3a*$^{+2}$ (N=7), and *Ube3a*$^{+4}$ (N=8). *Ube3a*$^{+4}$ samples included in the line C group are from line C *Ube3a*$^{+2}$ homozygotes. "C + E" *Ube3a*$^{+4}$ samples comprise line C/line E *Ube3a*$^{+2}$ heterozygotes. One-way ANOVA. (**C**) Linear detection of increasing *Rpl22* (left panel) and *Ube3a* (middle panel) mRNA levels across an 8-fold range of cDNA input (right panel). (**D**) Mean ± SEM whole-brain *Ube3a* transcript levels as reported in main Fig. 1, broken out by specific *Ube3a*^{OE} lines, as described for panel B. (**E**) Box plots of ratios of whole-brain *Ube3a* transcript and UBE3A protein levels for corresponding samples from panels D and B, respectively. Whiskers represent 5-95% confidence intervals. One-way ANOVA. (**F**) Schematic describing *Ube3a* exon structure and primer pairs designed to amplify total *Ube3a* transcript (Universal-1 and Universal-2) and *Ube3a* isoform 2 (*Ube3a-Iso2*) and *Ube3a* isoform 3 (*Ube3a-Iso3*) individually. (**G**) Mean ± SEM whole-brain *Ube3a* transcript levels, normalized to *Rpl22* and broken out by specific *Ube3a*^{OE} lines, for both universal and isoform-specific assays (as per panel F). Data from panels C-E and main Fig. 1 were produced using the Universal-1 assay. ns, *non-significant*.

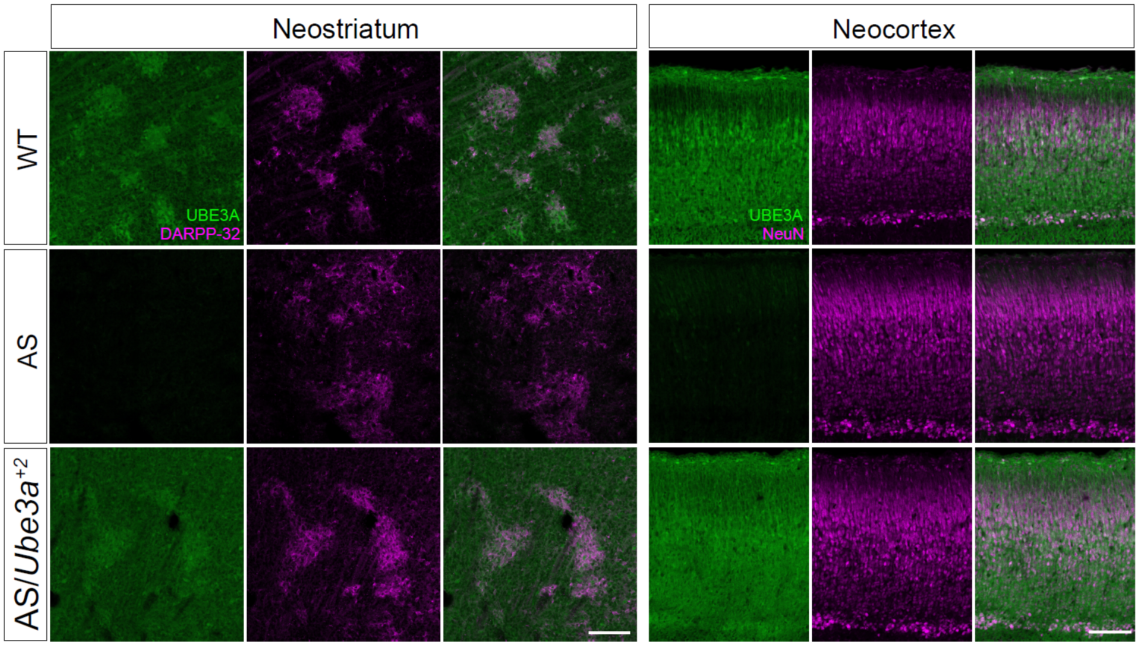

Figure S3. Neonatal patterns of UBE3A expression are conserved in *Ube3a*^{OE} mice. UBE3A immunofluorescence staining (green) in the neostriatum and neocortex of P1 WT, AS, and AS/*Ube3a*$^{+2}$ double-mutant mice. Counterstaining (magenta) for DARPP-32 and NeuN labels striasomal compartments in the neostriatum and mature neurons in the neocortex, respectively. Scale bars = 100 μm.

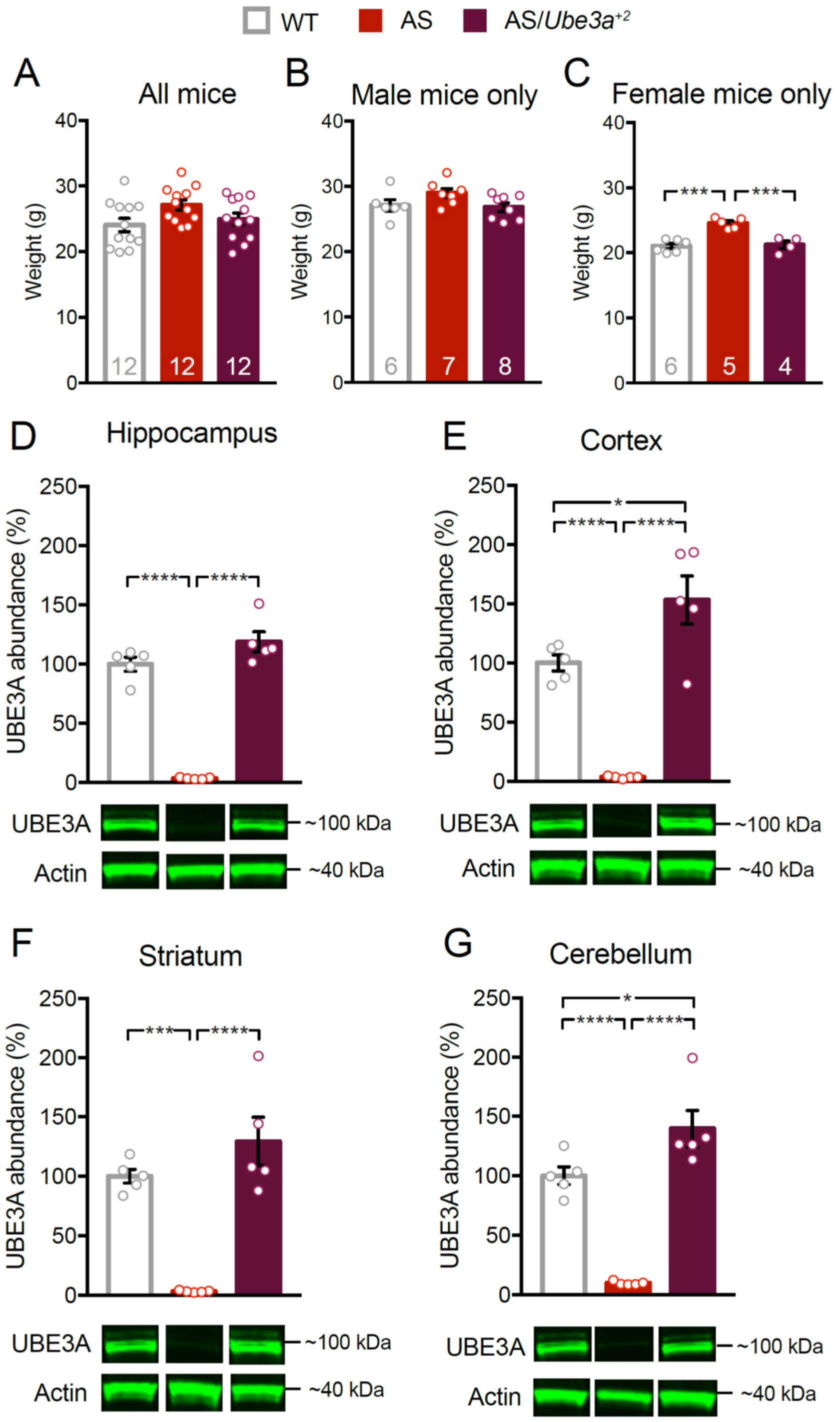

Figure S4. Body weight and UBE3A protein expression levels in mice used in Figure 3 behavioral experiments. (**A-C**) Mean ± SEM body weight of wild-type (WT) (N=12—6 male, 6 female), *Ube3a*$^{m-/p+}$ (AS) (N=12—7 male, 5 female) and *Ube3a*$^{+2}$ x *Ube3a*$^{m-/p+}$ (AS/*Ube3a*$^{+2}$) (N=12—8 male, 4 female) mice in aggregate (A) and broken out by sex for males (B) and females (C). Sample sizes are indicated in each bar. Representative blots and quantification of UBE3A protein levels (relative to Actin) in the (**D**) hippocampus, (**E**) cortex, (**F**) striatum, and (**G**) cerebellum of WT, AS, and AS/*Ube3a*$^{+2}$ mice. Average WT UBE3A expression is set at 100%. N=5 per group. One-way ANOVA with Tukey's *post hoc* test. **P*<0.05, ****P*<0.001, *****P*<0.0001.

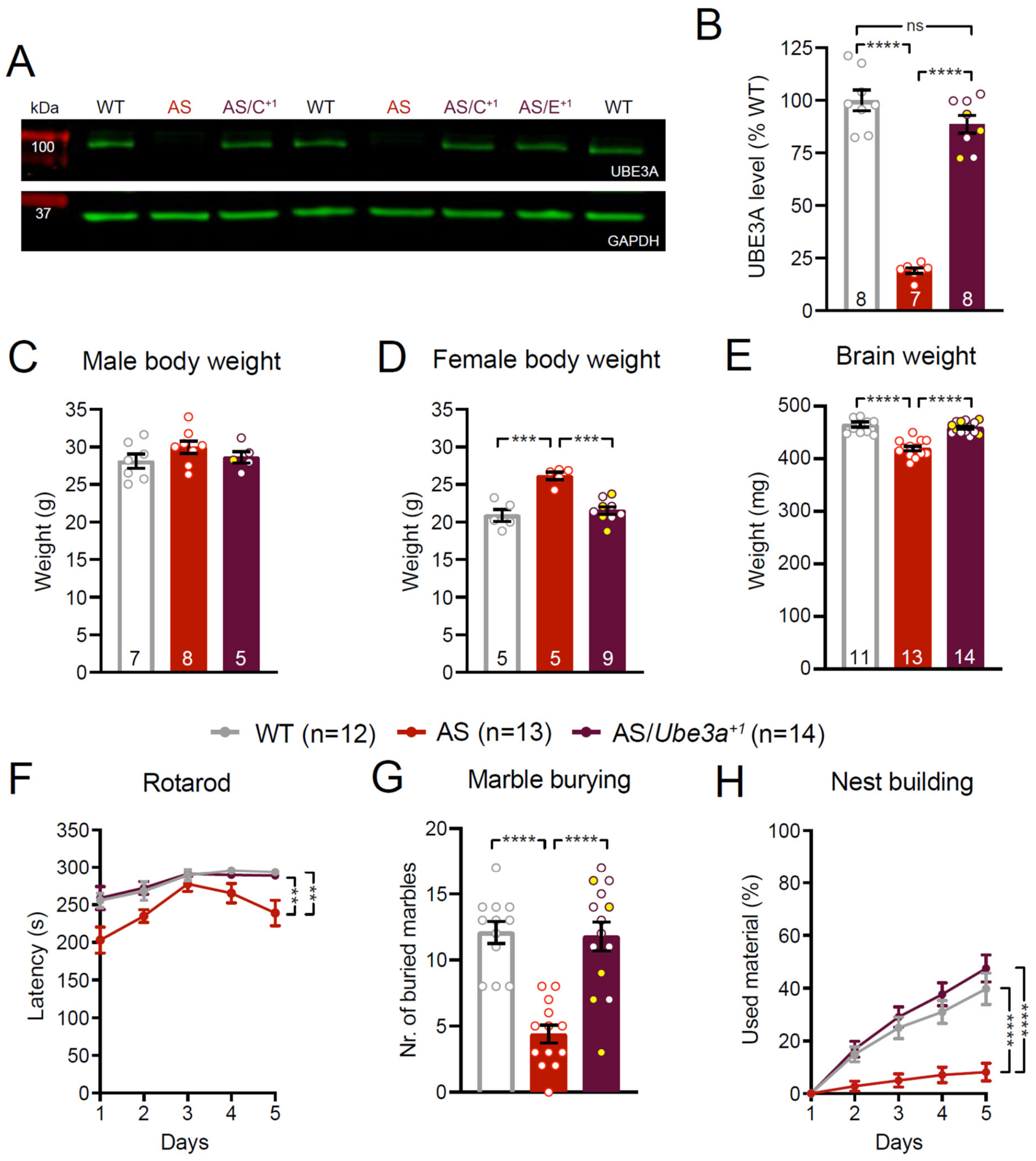

Figure S5. Transgenic UBE3A protein rescues behavioral phenotypes observed in AS mice. (**A**) Representative Western blot (WB) depicting immunofluorescent detection of bands corresponding to total whole-brain UBE3A and the loading control protein, GAPDH. Bands at left (red) correspond to the molecular weight marker. (**B**) Mean ± SEM UBE3A WB immunofluorescence intensities as determined from wild-type (WT), *Ube3a*$^{m-/p+}$ (AS), and *Ube3a*$^{m-/p+}$ x *Ube3a*$^{+1}$ (AS/*Ube3a*$^{+1}$) whole-brain lysates. Welch's ANOVA, Dunnett's *post hoc*. (**C-D**) Mean ± SEM body weight of WT, AS, and AS/*Ube3a*$^{+1}$ mice broken out by sex for males (C) and females (D). One-way ANOVA, with Tukey's *post hoc* test. (**E**) Mean ± SEM postmortem brain weight for WT, AS, and AS/*Ube3a*$^{+1}$ mice. One-way ANOVA, with Tukey's *post hoc* test. (**F-H**) Group performance of WT, AS, and AS/*Ube3a*$^{+1}$ mice for fall latency on the standard rotarod task (F, repeated measures two-way ANOVA, with Tukey's *post hoc* test), marbles buried (G, one-way ANOVA, with Tukey's *post hoc* test), and nest building (H, repeated measures two-way ANOVA, with Tukey's *post hoc* test). White-filled and yellow-filled circles represent data from AS/*Ube3a*$^{+1}$ mice from line C and line E matings, respectively. Sample sizes unless otherwise specified in bar graphs: WT, N=12; AS, N=13; AS/*Ube3a*$^{+1}$, N=14. **P<0.01, ***P<0.001, ****P<0.0001.

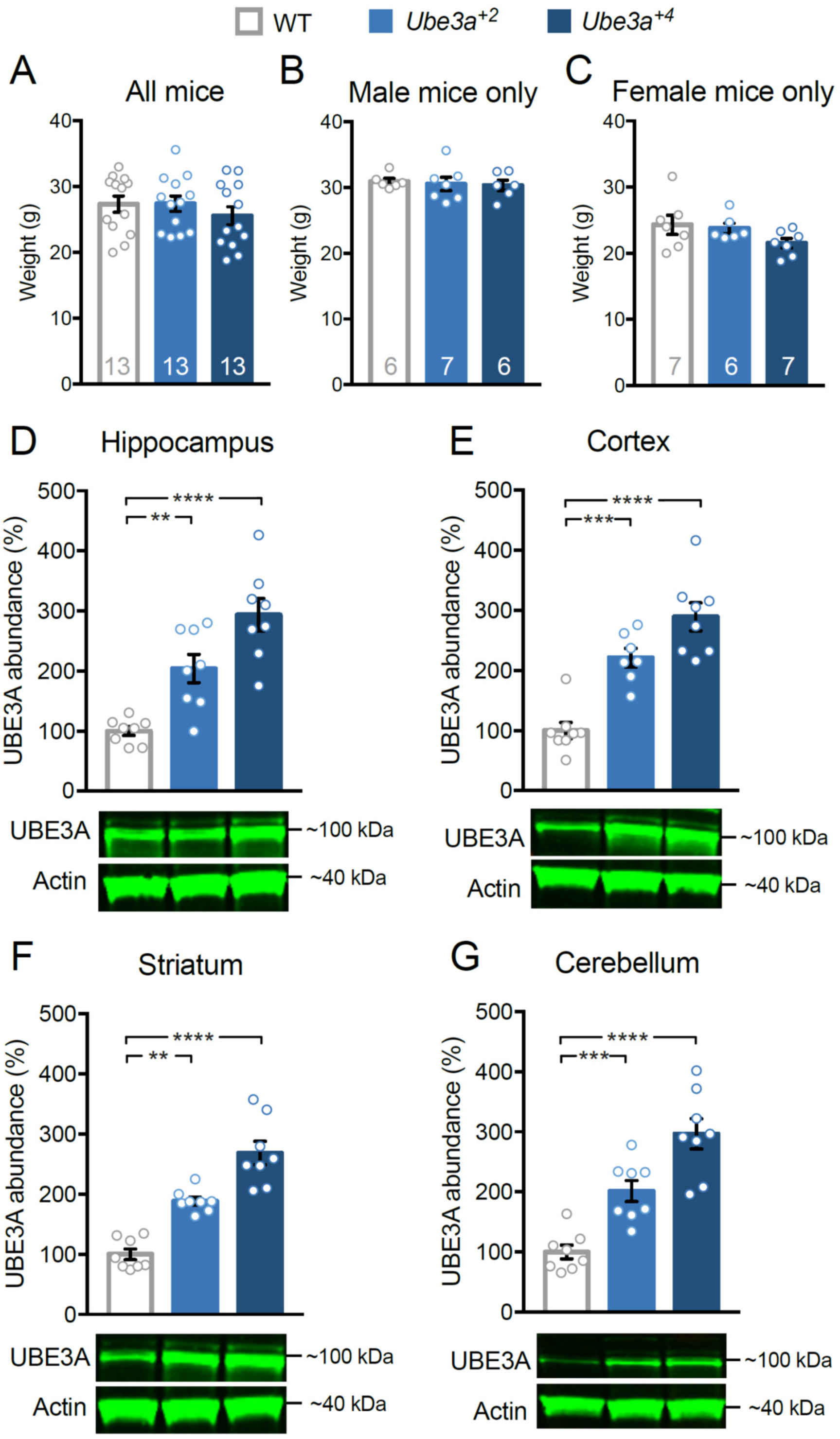

Figure S6. Body weight and UBE3A protein expression levels in mice used in Figure 4 behavioral experiments. (**A-C**) Mean ± SEM body weight of wild-type (WT) (N=13—6 male, 7 female), *Ube3a*$^{+2}$ (N=13—7 male, 6 female) and *Ube3a*$^{+4}$ (N=13—6 male, 7 female) mice in aggregate (A) and broken out by sex for males (B) and females (C). Sample sizes are indicated in each bar. Representative blots and quantification of UBE3A protein levels (relative to Actin) in the (**D**) hippocampus, (**E**) cortex, (**F**) striatum, and (**G**) cerebellum of WT, *Ube3a*$^{+2}$ and *Ube3a*$^{+4}$ mice. Average WT UBE3A expression is set at 100%. N=8 per group. One-way ANOVA with Tukey's *post hoc* test. $^{**}P<0.01$, $^{***}P<0.001$, $^{****}P<0.0001$.

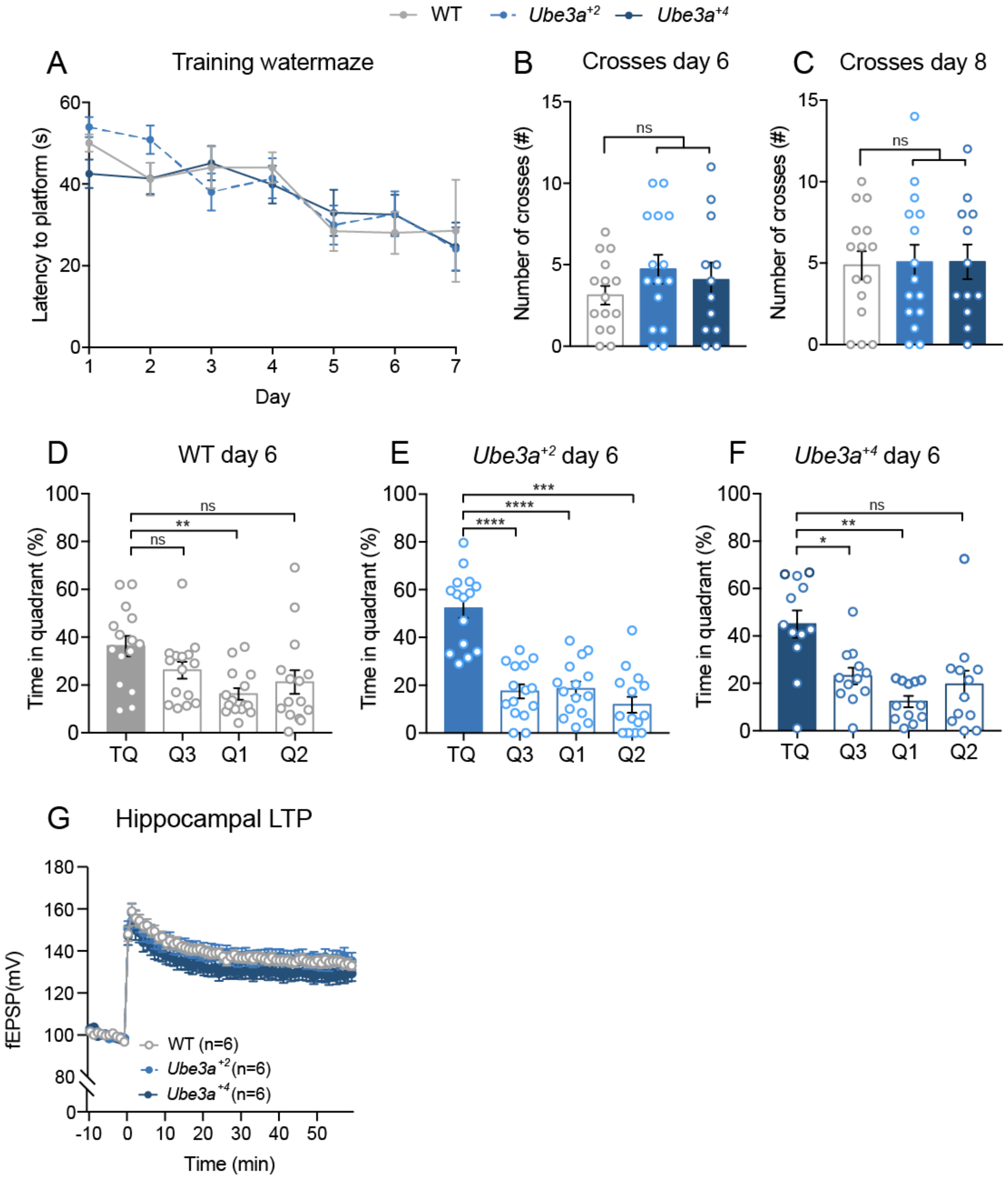

Figure S7. Water maze training, platform crosses, and performance on day 6; hippocampal LTP. (**A**) Mean ± SEM platform escape latencies for wild-type (WT) (N=15), *Ube3a*$^{+2}$ (N=15), and *Ube3a*$^{+4}$ (N=12) mice during training. Repeated measures two-way ANOVA. (**B-C**) Mean ± SEM platform crosses by genotypic group (day 6 and day 8). Kruskal-Wallis with Dunn's *post hoc* test. (**D-F**) Spatial memory acquisition as determined from day 6 testing in the Morris water maze paradigm. Mean ± SEM graphing of average time spent by WT (D), *Ube3a*$^{+2}$ (E), and *Ube3a*$^{+4}$ (F) mice in maze quadrants Q1, Q2, Q3, and the target quadrant-containing platform (TQ). Repeated-measures one-way ANOVA, with Dunnet's *post hoc* test. (**G**) Hippocampal LTP measured in sagittal slices of WT (N=6), *Ube3a*$^{+2}$ (N=6), and *Ube3a*$^{+4}$ (N=6) mice. The recorded fEPSP was compared between the three genotypes. Repeated-measures two-way ANOVA, with Bonferroni *post hoc* test. ns, *non-significant*, *P<0.05, **P<0.01, ***P<0.001, ****P<0.0001.

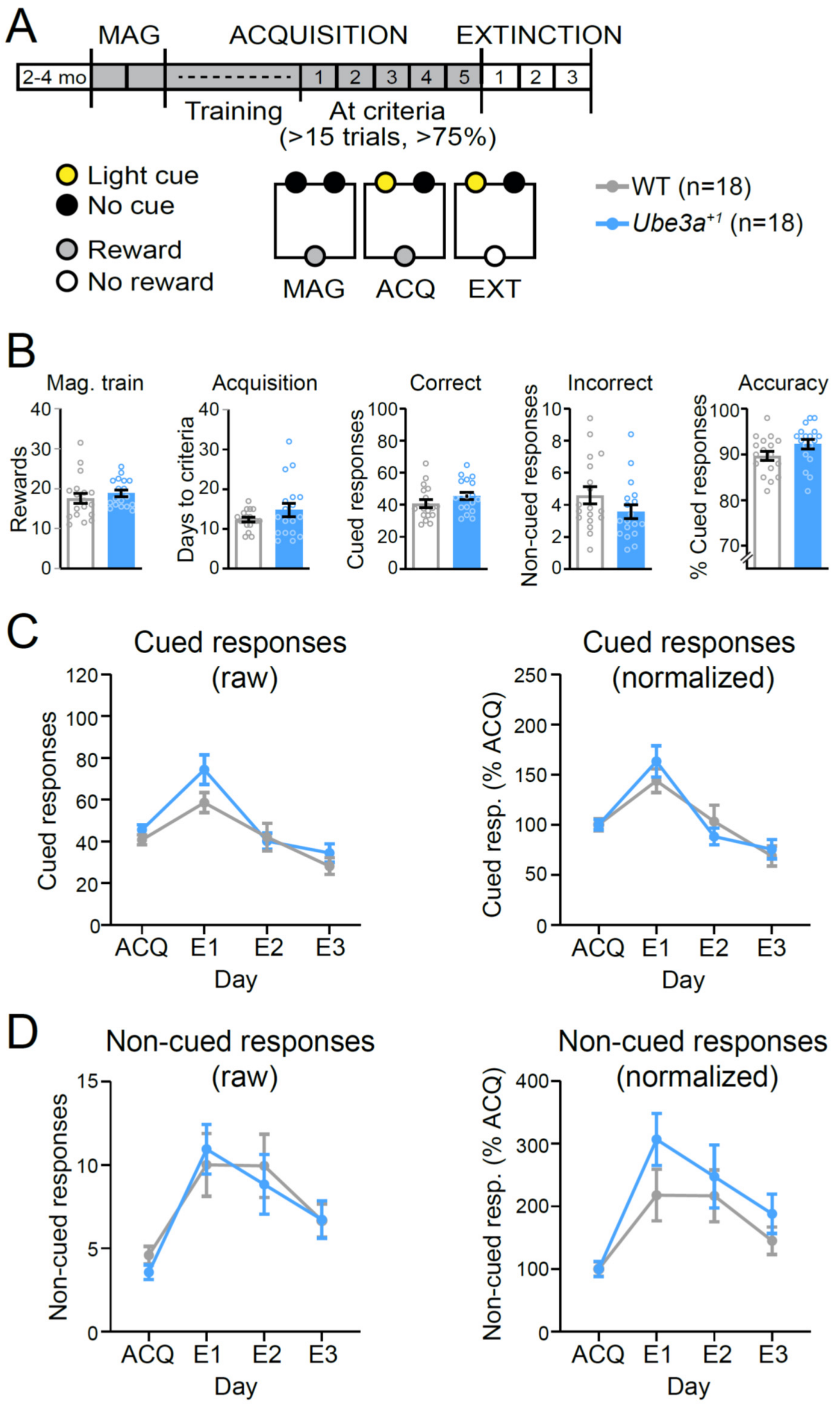

Fig S8. *Ube3a*$^{+1}$ mice exhibit normal operant extinction. (**A**) Schematics of the experimental timeline for the magazine training (MAG), acquisition (ACQ), and extinction (EXT) tests (top) and of the operant conditioning chambers (bottom). Behavioral chambers contained a food magazine and two nose-poke apertures. Gray indicates the presence of food reward (MAG, ACQ), and white indicates the absence of reward (EXT). Yellow indicates the presence of the light cue—black, its absence. (**B**) Left to right: graphs of mean ± SEM rewarded nose-pokes during MAG training, days to reach operant acquisition criteria, and raw correct and incorrect responses at ACQ criteria with computed response accuracy (proportion of correct responses to the cued aperture). Unpaired two-tailed *t* test. (**C-D**) Graphs of mean ± SEM cued (C) and non-cued (D) responses over the last 5 days of ACQ and during each day of EXT. Raw responses are plotted in the graphs on the left. Normalized responses (to the group means of cued responses during the last 5 days of ACQ) are plotted in the graphs on the right. Two-way ANOVA, Bonferroni's *post hoc*. WT (N=18), *Ube3a*$^{+1}$ (N=18).

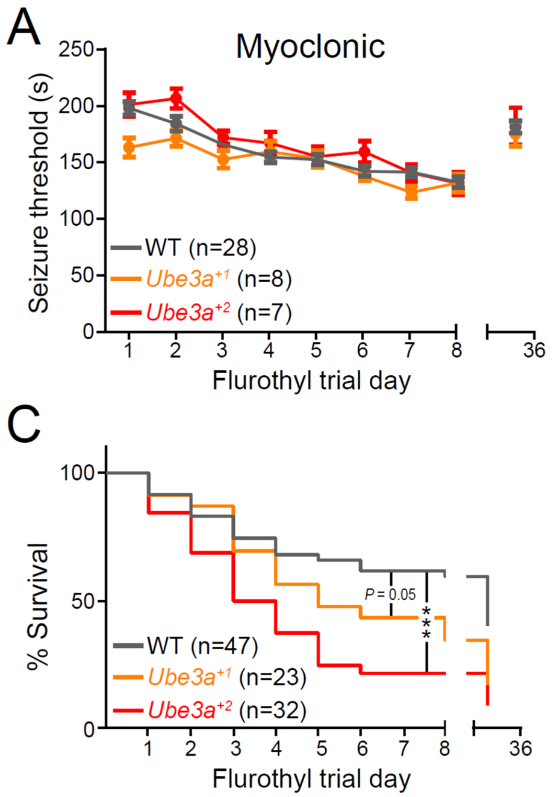

Figure S9. Enhanced susceptibility to seizure-associated death is also observed in line C *Ube3a^{OE}* mice. (**A-B**) Graphs of mean ± SEM latencies to myoclonic (A) and generalized (B) seizure depicting changes in seizure threshold throughout flurothyl kindling and rechallenge. Data represent mice surviving all 8 days of flurothyl kindling. Two-way repeated measures ANOVA, Tukey's *post hoc*. WT (N=28), *Ube3a^{+1}* (N=8), *Ube3a^{+2}* (N=7) (**C**) Group survival for all flurothyl-kindled mice. Survival curves were compared with the Log-rank (Mantel-Cox) test. WT (N=47), *Ube3a^{+1}* (N=23), *Ube3a^{+2}* (N=32) ***P < 0.001.

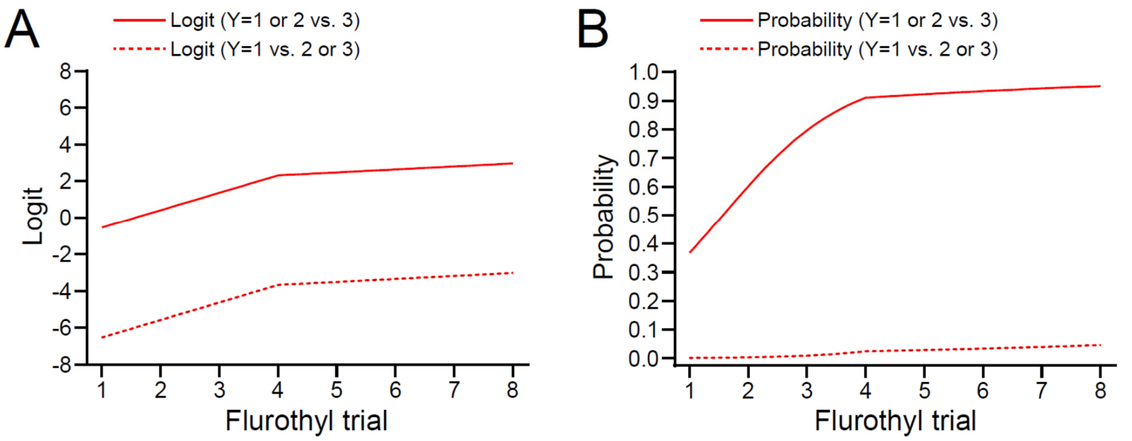

Figure S10. Supplemental analysis of changes in seizure severity during flurothyl kindling (in support of Figure 7C). (A) "Linear in the logits" plot where the logit is the natural log of the odds, and the odds is the probability of the outcome (i.e., seizure severity) divided by one minus the probability. (**B**) Plot of the back-transformation of the logits into the probability of outcome as a function of the flurothyl kindling trial. Figure 7C indicates that seizure severity increases over the first four flurothyl trials, stabilizes for the remaining four kindling trials, and that this pattern is similar for the three groups. To formally test this, we used a mixed effects generalized linear model (GLM) in which trial is nested within mouse (a random within-subjects factor) and genotypic group membership is a characteristic of each mouse (a fixed between-subjects factor). Because of sparseness in scores across the original one to seven ordinal scale, we created a trichotomous measure in which a value of one represented "3" on the original scale, a value of two represented "4", and a value of three represented "5 or more". Pooling over all observations, 20% were equal to one, 77% were equal to two, and 3% were equal to three. Given the trichotomous outcome, we defined the GLM using a standard multinomial response distribution and cumulative logit link function estimated using maximum likelihood with numerical quadrature (nine points of integration). Given the observed pattern of change over time, we used a piecewise linear spline in which the two pieces jointly defined a linear trajectory, the first leading up to the knot point at the fourth trial and the second continuing from the fourth trial forward (e.g., (1)). As such, the first piece was coded -3, -2, -1, 0, 0, 0, 0, 0 and the second piece 0, 0, 0, 0, 0, 1, 2, 3. The intercept was defined to be the model-implied mean of the outcome at the transition point (i.e., trial four). With a trichotomous outcome there are two intercept terms; the first reflects the probability of a score greater than 1, and the second the probability of a score greater than 0. The initial model was estimated including both linear pieces and genotypic group membership. Both the main effect of group ($F[2,324]=.46$, $p=.63$) and the interaction between group and change in outcome over time ($F[2,324]=.65$, $p=.52$) were non-significant; the effect of group was thus removed from the model to better clarify pattern of change over time. Consistent with the pattern observed in figure panels A and B, the slope of the first linear piece was increasing and significant ($b_1=.954$, $p<.0001$) and the slope of the second linear piece did not significantly differ from zero ($b_2=.162$, $p=.23$). In other words, there was a significant linear increase in the endorsement of higher values on the outcome over the first four trials (the odds of endorsing a higher value on the outcome increases by a factor of 2.6 per trial), but there was no observable increase subsequent to trial four. For completeness, we re-estimated the model using a standard linear mixed-effects model that assumes continuity of the original ordinal outcome measure; no substantive group differences in trajectory parameters were found.

Table S1. Genomic summary of conditional *Ube3a*^{OE} lines.

Line	Germline Transmission	Integration Site	Sublines	*Ube3a* copies	
				Transgenic	Total
A#	Yes	Not Mapped	Not Generated	2	4
B#	No	–	–	–	–
C*	Yes	Chr3	*Ube3a*$^{+1}$	1	3
			Ube3a^{+2}	2	4
D#	Yes	–	–	–	–
E*	Yes	Chr3	*Ube3a*$^{+1}$	1	3
			Ube3a^{+2}	2	4
F	Yes	Not Mapped	N/A	1	3

*Used in this study; #Discontinued; N/A, not applicable.

Table S2. Primer sets used in TLA analysis.

Primer Set	Transgene Binding Position	Direction	Sequence*
1	33169	F	GTATGTGTCCTGTTCCTCTG
	33328	R	CCCTTAATCTTGAAGCAAGC
2	75683	F	CTCTCCACTAGTCTCCTCTT
	75847	R	AAACTAAGGGAACTATGCCA
3	137742	F	TCCTAATGCTAAGCGTGATG
	138172	R	CATATTCCATACACGCAAGC

*All primers also anneal to homologous sequences in mouse chr7.

Table S3. ddPCR primer and probe sequences.

Target	Primers/Probes	Sequence
Ube3a gene and *Ube3a*^{OE} transgene (intron 3/exon 3)	F	AGAACGCTACTACCATCAGT
	R	GAGGATCACAGAGTTTTGCA
	TaqMan probe	CGTGCAGGCCTCATTTCCACAGCCC
Ube3a^{OE} transgene (loxP)	F	AGCTTTCTTGTACAAAGTGG
	R	GGTCTAAGGGCCTATGAATT
	TaqMan probe	TGTATGTCTTCATGTAGCCGCCTCTGC
Tfrc gene (exon 17)	F	GTTCCTCAGCTCAACCAAAT
	R	GAACTGGTTCAGATCCTTCA
	TaqMan probe	AGCCACTTCCGCTGCTGTACGAACC
Total *Ube3a* mRNA "Universal-1" (exon 5/exon 6)	F	TGAACAAGAAGGAAGGAAAAGA
	R	CAATTCCTCCTTTGTGTGCTG
	TaqMan probe	CCGAAAGCTCAGAACCAGTGCCTCAGCA
Total *Ube3a* mRNA "Universal-2" (exon 4/exon 5)	F	TCCCAGTCTGAGGACATTGA
	R	TGGACAGGAGGCACAAAACT
	TaqMan probe	CGTGCAGGCCTCATTTCCACAGCCC
Ube3a isoform 2 mRNA "*Ube3a-Iso2*" (exon 3/exon 3-4)	F	CTGGTGTGACAACCTGTTTT
	R	CTGGTGATCTTTTACAAGCTGT
	TaqMan probe	TGTTATCACCCTGATGTCACCGAATGGCC
Ube3a isoform 2 mRNA "*Ube3a-Iso3*" (exon 1-4/exon 5)	F	CGAGGACAGATCACCAGG
	R	GCGTTCTATTAGATGCTTTGC
	TaqMan probe	CCCAGTCTGAGGACATTGAAGCTAGCCG
Rpl22 mRNA (exon 2/exon 3)	F	CTGCACTCACCCTGTAGAAG
	R	AGTGACAGTGATCTTGCTCT
	TaqMan probe	CCGCCGAGGTTGCCAGCTTTCCC

Table S4. Genotyping primers.

Line	Target	Primer	Sequence
Ube3a^{OE} (line C)*	3.2 kb chr3 deletion: *Smad9* exon 3 and *E. coli* breakpoint sequences	*Smad9* F	GGTACTCATCCAAAAGCCAAA
		Smad9 R	GCTTGCATAACCCCTTCAGA
		E. coli R	ACTGACCGTAACCATGTCAGA
Ube3a^{OE} (line E)*	Chr3 rearrangement: *E. coli* genomic sequence (pos. 2480152) fused to chr14 genomic sequence (pos. 115044966)	Chr14 F	TCATTTCATTTTCGTGTTTGC
		E. coli F	GATGTCCAGCCCGTATCGT
		Chr14 R	TGGTAAGAACAGTCCAAGGATAG
Ube3a$^{m–/p+}$	Region spanning deleted *Ube3a* exon 5	WT F	GCTCAAGGTTGTATGCCTTGGTGCT
		Mut F	TGCATCGCATTGTGTGAGTAGGTGTC
		WT R	ACTTCTCAAGGTAAGCTGAGCTTGC
Ube3a$^{mE113X/p+}$	Region spanning PTC in exon 5	WT F	GTTGTCAGTCAGAAGCAAGTT
		Mut R	TAAGTTACGTCCCTAAGCAGACG
		WT R	TTCTCCTTTTACCACAGTCCC
Ube3a$^{F/+}$ *Ube3a*$^{F/F}$	Region spanning floxed *Ube3a* exon 7	F	AAAATTGGGTATGCGAGCTG
		R	GGGGTCTAAGGGCCTATGAA
Gad2-Cre *NEX-Cre*	Cre recombinase	F	GATGGACATGTTCAGGGATCGCC
		R	CTCCCACCGTCAGTACGTGAGAT

*Primers can be used to amplify target regions in both *Ube3a*$^{+1}$ and *Ube3a*$^{+2}$ sublines.

Supplemental methods

Generation of Ube3a overexpression (Ube3a^{OE}) lines. BAC transgenic conditional *Ube3a* overexpression mice (*Ube3a* OE) were generated in the UNC Animal Models Core facility. Briefly, a Ube3atm1a_KOMP_W (*Ube3a^{KO1st}*) targeting cassette, which was generated by the trans-NIH Knockout Mouse Project (KOMP) and obtained from the KOMP repository (www.komp.org), was recombineered into the 162 kb *Ube3a* BAC clone RP24-178G7 (pTARBAC1 vector backbone) according to established procedures. Successfully recombineered BAC clones were identified by PCR and transformed into bacteria with inducible FLP recombinase expression to ultimately generate the conditional *Ube3a^{OE}* BAC construct. *Ube3a^{OE}* BAC was purified on anion exchange columns according to a modified Bimboim-Doly method (2), and BAC integrity was confirmed using pulse-field electrophoresis. Purified *Ube3a^{OE}* BAC was linearized via PI-SceI restriction digest prior to pronuclear microinjection of C57BL/6J fertilized eggs, which were subsequently implanted into pseudopregnant female mice. Six independent conditional *Ube3a^{OE}* founder offspring (named A–F) were confirmed by PCR analysis of tail DNA. Two parental conditional *Ube3a^{OE}* lines—C and E—were identified for use in this study following verification of germline transmission, mapping of chromosomal integration via TLA (see below), assessment of transgene copy number via ddPCR (see below), and confirmation of transgenic *Ube3a* expression. Both parental lines were confirmed to express two transgene copies (*Ube3a^{+2}*). Line C *Ube3a^{+2}* mice and line E *Ube3a^{+2}* mice were each subsequently crossed to highly efficient FLPo deleter mice (RRID:IMSR_JAX:011065) to recombine the remaining FRT sites and collapse transgenic loci to a single conditional *Ube3a^{OE}* copy, thereby establishing stable line C *Ube3a^{+1}* and line E *Ube3a^{+1}* sublines upon breeding out the FLPo allele in the subsequent generation. See Table S1 for a summary of conditional *Ube3a^{OE}* lines.

Targeted locus amplification (TLA) and next generation sequencing-based mapping of Ube3a^{OE} integration sites. Viable frozen mouse spleen cells were used and processed by Cergentis (Utrecht, NL) according to an established TLA protocol (3). 3 primer sets were designed on the *Ube3a^{OE}* transgene (Table S2) and were employed in individual TLA

amplifications. PCR products were purified and library prepped using the Illumina Nextera flex protocol and sequenced on an Illumina sequencer (Illumina, San Diego, CA). Reads were mapped using BWA-SW (4)(version: 0.7.15-r1140; settings: bwasw-b7), and subsequently aligned to transgene and host genome (*Mus musculus*, mm10) sequences. Chromosomal integration sites were detected based on coverage peaks in the genome as well as the identification of fusion-reads between the transgenic and host genome sequences. Line C chromosomal integration mapped to chromosome 3 at position 54,784,876–54,788,068, where there occurred a 3.2 kb deletion of *Smad9* exon 3 and the co-integration of 39.5 kb of E. coli genomic sequence. Line E integration also mapped to chromosome 3, approximately at position 106,000,000, where there occurred a structural rearrangement to chr14 and the co-integration of 100 kb of *E. coli* genomic sequence.

ddPCR-based analysis of Ube3a gene copy number in Ube3a^{OE} mice. Droplet digital PCR (ddPCR, QX200 platform, Bio-Rad, Hercules, CA) was used for analysis of both genomic DNA (gDNA) copy number and mRNA transcript levels (see below). 20 ng of PstI- and SspI-digested gDNA served as the input for ddPCR reactions to assess total and transgenic (i.e., loxP sequence-containing) copies of *Ube3a*, respectively. Each assay comprised two primer/probe sets: one designed to detect a target *Ube3a* genomic sequence, the other the 2-copy reference gene, *Tfrc*. For all ddPCR experiments described in this study, primers were prepared at a final concentration of 900 nM and accompanying probes at 250 nM. Target probes were labeled with 5' 6-Carboxyfluorescein (6-FAM) fluorophores; reference probes were labeled with 5' single-isomer fluorescein (HEX) fluorophores; all probes utilized 3' Iowa Black quenchers. Sequences and design details for each primer/probe set can be viewed in Table S3.

Established mouse lines. These studies incorporated the following established mouse lines: *Ube3a*$^{m-/p+}$ (5)(RRID:IMSR_JAX:016590) and *Ube3a*$^{mE113X/p+}$ Angelman syndrome (AS) model mice (6)(RRID: MGI: 5911277); Heterozygous (*Ube3a*$^{F/+}$) and homozygous (*Ube3a*$^{F/F}$) floxed *Ube3a* knock-in mice (7).

Breeding and husbandry. All mice were housed on a 12:12 light:dark cycle with *ad libitum* access to food and water. Male and female littermates were included in experimental groups at equivalent genotypic ratios. All mice used in experiments were born to breeders maintained on a congenic C57BL/6J background with three notable exceptions. First, to supply AS/*Ube3a*$^{+2}$ rescue experiments (Figures 3A-3F and S4A-S4G), C57BL/6J heterozygous *Ube3a*$^{+2}$ male mice (from line E) were bred with 129sv female *Ube3a*$^{m+/pE113X}$ (AS) mice, yielding wild-type (WT), AS, and AS x *Ube3a*$^{+2}$ (AS/*Ube3a*$^{+2}$) offspring on an F1-hybrid 129sv-C57BL/6J background. Second, for a subset of UBE3A overexpression experiments (Figures 4A-4F, S6A-S6G, and 5A-G), line E heterozygous *Ube3a*$^{+2}$ mice backcrossed 1 or 2 generations onto the 129sv background (from C57BL/6J) were bred with C57BL/6J heterozygous *Ube3a*$^{+2}$ mice to produce WT, heterozygous *Ube3a*$^{+2}$, and homozygous *Ube3a*$^{+4}$ offspring. Third, all audiogenic seizure experiments were performed with mice backcrossed at least 5 generations onto the 129sv background.

Mouse genotyping was performed at 4-7 days of age, with re-genotyping upon the completion of experiments to confirm identity. See Table S4 for genotyping information. With the exception of mice used for immunofluorescence studies, all mice were 5-33 weeks of age at the start of the experimentation. Before and during experiments, 2-5 sex-matched mice were group-housed in individually ventilated cages, except during the nest building and forced swim tests, for which mice were individually housed. More detailed information regarding sex and age distribution per cohort can be found in Supplemental Data 3.

Tandem RT-ddPCR and Western blotting analysis of Ube3a/UBE3A overexpression. Tissue processing. Adolescent postnatal day (P)45-60 mice were anesthetized with isoflurane and sacrificed by acute decapitation. Brains were dissected into left and right cerebral hemispheres, immediately snap-frozen on liquid nitrogen, and stored at -80°C until later use in ddPCR and Western blotting experiments.

RT-ddPCR. For reverse transcriptase (RT)-ddPCR assays to assess levels of *Ube3a* mRNA, 2.5 ng of cDNA input was used unless otherwise specified. cDNAs were reverse transcribed via iScript random priming (Bio-Rad) from whole-brain RNAs extracted using the

RNeasy Mini Kit (Qiagen, Germantown, MD). RT-ddPCR assays comprised two primer/probe sets: one against *Ube3a* transcript (universal or isoform-specific), the other against the reference transcript *Rpl22* (Table S3).

Western blotting. Frozen tissue was thawed on ice and subsequently homogenized (3-5 rounds, 2-3 seconds each with a hand-held homogenizer: Tissue Tearor, Daigger Scientific, Hamilton, NJ) in SDS lysis buffer composed of 25 mM Tris-HCl pH=7.5, 1% SDS, and 1/100 protease inhibitor cocktail (Sigma-Aldrich, P8340). Homogenized samples were cleared by centrifugation for 15' at 20,000 x g, after which the protein concentration was determined using BCA reagent kit (Pierce, 23227).

Approximately 15 µg of total protein lysate per sample was separated by SDS–PAGE (Biorad, #456-1093) and transferred onto 0.2 µm nitrocellulose membranes (Biorad, #170-4159). Membranes were blocked for 1 h at room temperature in 5% (w/v) powdered milk solubilized in TBS-T: Tris-buffered saline (10 mM Tris-HCl, pH 8.0, 150 mM NaCl) containing 0.1% Tween-20 (Sigma P1379). Blocked membranes were washed three times in TBS-T and incubated at 4°C overnight with primary antibody diluted in TBS-T + 2% w/v milk. The day after, membranes were washed 3 times for 10 minutes with TBS-T and incubated with the secondary antibody diluted in TBS-T + 2% w/v milk for 1 h. Finally, membranes were washed 3 times for 10 minutes with TBS and analyzed by measuring infrared fluorescence (Li-Cor Biosciences, Odyssey CLx). Protein band intensities were quantified and analyzed using ImageJ (RRID:SCR_003070) (8). The following primary and secondary antibodies were used: 1:1,000 mouse anti-UBE3A (Sigma, 1404508, RRID:AB_10740376), 1:10,000 mouse anti-GAPDH (Millipore, MAB 374 RRID:AB_ 2107445), and 1:7,500 donkey anti-mouse 800CW (LI-COR Biosciences Cat# 926-32212, RRID:AB_621847).

Immunohistochemistry - Tissue processing. P1 and P25 mice were anesthetized with sodium pentobarbital (60 mg/kg) prior to transcardial perfusion with phosphate-buffered saline (PBS, pH 7.5), immediately followed by phosphate-buffered 4% paraformaldehyde, pH 7.3. Perfused brains were postfixed overnight at 4°C in the same fixative before sequential 12 h incubations in 10%, 20%, and 30% sucrose in PBS. Cryoprotected brains were frozen on dry

ice and cut into 40-μm-thick sections with a sliding microtome. Prior to immunofluorescent staining, sections were stored at −20°C in a cryopreservative solution (by volume: 45% PBS, 30% ethylene glycol, 25% glycerol).

Immunofluorescent staining. Brain sections were rinsed several times in PBS before blocking in PBS plus 5% normal goat serum and 0.2% Triton X-100 (NGST) for 1 h at room temperature. Blocked sections were subsequently incubated in primary antibodies diluted in NGST for 48 h at 4°C. Several rinses in PBS containing 0.2% Triton X-100 (PBST) followed, just before incubation in secondary antibodies (also diluted in NGST) for 1 h at room temperature. In most experiments, 4′,6-diamidino-2-phenylindole (DAPI; Thermo Fisher Scientific, D1306) was also included at a concentration of 700 ng/mL for nuclear counterstaining. Primary antibodies and reagents used included 1:500 mouse anti-NeuN (Millipore, MAB377, RRID:AB_10048713), 1:1,000 mouse anti-UBE3A (Sigma, 1404508, RRID:AB_10740376), and 1:500 rabbit anti-DARPP-32 (Millipore, AB10518, RRID:AB_10807019). The following secondary antibodies (Thermo Fisher Scientific) were used at 1:500: Alexa Fluor-488 goat anti-mouse IgG2a (A21131, RRID:AB_141618), Alexa Fluor-647 goat anti-mouse IgG1 (A21240, RRID:AB_141658), and Alexa Fluor-568 goat anti-rabbit IgG (A11036, RRID:AB_143011). All brain sections compared in figures were stained within the same experiment under identical conditions. Images of immunofluorescently labeled brain sections were acquired with a Zeiss LSM 710 confocal microscope equipped with ZEN imaging Software (Zeiss, RRID:SCR_013672). Images for quantitative comparison were collected during the same imaging session using identical acquisition parameters.

Behavioral test-battery for Ube3a dose-sensitive behaviors. Methodology for the following behavioral tests (rotarod, open field, marble burying, nest building, forced swim, and audiogenic seizure induction) has been described in detail in a previous study of behavioral phenotypes in AS model mice (9), and is provided in brief below. A separate cohort of 129sv-backcrossed mice was used in the audiogenic seizure experiments.

Standard and reversed rotarod tests. Mice were trained to walk on an accelerating rotating rotarod. In the standard assay, the mice walked within chutes wide enough for them

to turn fully around, though they tended to walk almost exclusively in the forward direction. In the reversed rotarod, the chutes were narrowed, and mice were forced to walk backwards—a more challenging task. Over two 5-minute trials per day, administered on five consecutive days, the average time spent on the rotarod was calculated. Motor performance was expressed as latency to fall from the apparatus.

Open field test. Mice were placed in an open field with a 110-cm diameter. Locomotion was recorded for a period of 10 minutes, and the total distance travelled was compared between groups.

Marble burying test. Mice were put in a cage with an ample layer of bedding on top of which were placed 20 marbles in a 5 x 4 array. After 30 minutes, buried marbles (>50% covered in bedding material) were counted as a proxy for digging behavior. The total number of buried marbles was compared between groups.

Nest building. Individually housed mice were given a total of 11 grams of compressed extra-thick blotting paper, serving as nest material. Unused nest material was weighed over five consecutive days to gauge nest building progress.

Forced swim test. Mice were put in a cylindrical tank filled with water of 27°C for a total of 6 minutes. Mice were left to acclimatize for the first two minutes of the test. Floating time, defined as the percentage of time that mice were immobile, was measured during the remaining 4 minutes of the task.

Audiogenic-induced seizures. Mice were placed in a Macrolon cage (50 x 26 x 18 cm) fitted with a cage top made up of a metal grid. Audiogenic seizures were induced by vigorously and loudly (~100 dB) scratching a pair of scissors against the metal grid. This stimulus presentation was maintained for 20 seconds or until a tonic-clonic seizure occurred.

Three-chamber sociability experiment. WT and *Ube3a*$^{+4}$ mice (N=9) from the same cohort assessed by the *Ube3a* dose-sensitive behavioral battery (excluding audiogenic seizures) were subjected to a social interaction task, adopting the Crawley methodology (10). This 30-minute test measures a mouse's preference to interact with novel conspecifics versus familiar conspecifics or novel objects, and takes place within a social interaction box (63 x

42.2 x 22.2 cm) divided into three compartments—referred to as left (21 x 40.5 x 22.2 cm), center (17.5 x 40.8 x 22.2 cm), and right (21 x 40.5 x 22.2 cm)—using opaque dividers, each with a rectangular opening at the bottom that can be manually opened and closed. The left and right compartments house a weighted metal cup, under which a mouse can be placed. Mouse movement within the three-chamber arena is recorded using an infrared camera programmed with Bonsai (version 2.6.2, RRID:SCR_021512) software to enable automatic pausing when hatches are opened, and new mice are placed under the cups.

Experiments began with the habituation (10 minutes) of a test mouse to the center compartment. At the end of the habituation phase, an age- and gender-matched conspecific (i.e., a novel mouse) was placed under a cup in the left compartment; a corresponding cup in the right compartment was left empty to serve as a novel object. The dividers were then removed to initiate the sociability test, in which the test mouse was allowed to freely investigate all compartments of the box for a period of 10 minutes. In the subsequent social novelty test, a second age- and gender-matched conspecific (i.e., a new novel mouse) was placed under the previously empty cup in the right compartment, and the test mouse was allowed to freely investigate for an additional 10 minutes. The time spent in each compartment as well as in proximity to either conspecifics or the empty cup was analyzed using OptiMouse software (V3.0) and compared between genotypic groups. This set-up has been previously used to assess sociability in another mouse model (11).

Western blotting following Ube3a dose-sensitive and social behavioral assessment. Sample preparation. Following behavioral experiments (excluding audiogenic seizure induction), mice were sacrificed through cervical dislocation, brains were harvested, and brain regions (i.e., cerebellum, striatum, hippocampus, and cortex) were isolated via microdissection. All tissue was immediately snap-frozen in liquid nitrogen and stored at -80°C for later use. Frozen tissue was thawed on ice and subsequently sonicated (10 μm, 3 rounds of 4 seconds) in 2x Leammli buffer (25 mM Tris-HCl pH=7.5, 2% SDS + 1/100 protease inhibitor; Sigma-Aldrich, P8340). Samples were centrifuged for 5 minutes at 16,000 x g, after

which supernatant was taken, pellet discarded, and the protein concentration determined via BCA assay.

Western blot analysis. Approximately 20 µg of total protein lysate per sample was separated by SDS–PAGE (Biorad, #345-0123) and transferred onto 0.2 µm nitrocellulose membranes (Biorad, #170-4159). Membranes were subsequently immunoblotted using the same procedure described above for tandem ddPCR/Western blot analyses, but incorporating the following primary antibodies: 1:1,000 mouse anti-UBE3A (Sigma-Aldrich Cat# E8655, RRID:AB_261956), and 1:10,000 mouse anti-ACTIN (Millipore Cat# MAB1501R, RRID:AB_2223041).

Morris water maze. A separate *Ube3a*^{OE} cohort comprising WT, heterozygous *Ube3a*$^{+2}$, and homozygous *Ube3a*$^{+4}$ mice was bred for water maze testing to probe spatial learning deficits. These mice were extensively handled prior to the test (5 days, 2 minutes each day). During training over a period of 8 consecutive days, mice were given 2 trials (30-second intertrial interval) per day to find a platform (11 cm diameter) hidden just 1 cm beneath the surface of a pool (1.20 m diameter) filled with visually opaque warm water (25-26°C). Training trials were initiated by placing mice on the platform for 30 seconds, allowing them to form a spatial concept of the platform's location. Visual cues—colored shapes or patterns—placed around the pool facilitated spatial learning. Subsequently, the mouse was removed from the platform and placed into the pool from a pseudo-random starting position. Mice were allowed 60 seconds to find the platform. If successful, they were left on the platform for 30 seconds. If not, they were removed from the water and placed on the platform, also for 30 seconds. Each day, upon completion of the twice daily trials, mice were dried off and returned to their home cages. During all trials, the platform remained at the same position in the pool. On days 6 and 8, in addition to the training sessions, so-called probe trials were executed. As with training, probe trials began by placing mice on the platform for a period of 30-seconds. Next, mice were taken from the platform, and the platform itself was removed from the pool. Mice were then placed in the pool, opposite to where the platform was located, and their movement was recorded for 60 seconds using an infrared camera. The movement of

individual mice across and within the 4 quadrants of the pool was analyzed using EthoVision XT software (Noldus, RRID:SCR_004074) and compared between the different genotypes.

Fear conditioning. The fear-conditioning task was performed with the same cohort used for the Morris water maze task. Experiments were performed in a plexiglass testing chamber (26 x 22 x 18 cm; San Diego Instruments, San Diego, CA), equipped with a metal grid floor through which foot shocks were administered. Mouse movement was monitored with a CCD camera installed in front of the chamber. This setup was housed within a soundproof cubicle illuminated with white light. At the start of each experiment, mice were placed into the chamber and the soundproof cubicle was closed. Movement recorded during the first 150 seconds was used to establish baseline levels of mobility and immobility. Subsequently, a 2-second shock (0.8 mA) was administered through the metal grid floor, followed by a second shock of equal duration and intensity 30 seconds later. After a total elapsed time of 210 seconds, mice were returned to their home cages. Context-dependent learning was evaluated both 24 hours and 28 days later by reintroducing mice to the same testing chamber for 150-second sessions and recording their movement in the absence of shock administration. Freezing behavior, an indicator of fear in mice, was analyzed by SDI Photobeam Activity System software and freezing times were compared between the different genotypes tested. The sensitivity of this and the Morris watermaze task in our lab has been previously established (12–15).

Operant acquisition and extinction. Operant acquisition and extinction were measured in independent cohorts of *Ube3a*^{OE} mice using previously established methods (16). Mice were food restricted for the duration of experiments, but were allowed 1.5 hours of unrestricted feeding following each testing session. All experiments were performed during the light phase at the same time each day. Two of five available nose-poke apertures in the modular operant conditioning chambers (MED Associates, ENV-307W) were used for testing; the remaining three were covered. First, mice were trained (two 10-minute sessions on consecutive days) to receive a food reward (20 mg dustless precision pellets, Bio-Serv) every time they nose-poked into the food magazine ["magazine training" (MAG)]. During operant acquisition (ACQ; 15-

minute sessions), one of the two apertures was illuminated at a time, and mice received a reward only after a nose-poke into the illuminated (“cued”) aperture. “Non-cued” responses were defined as a nose-poke into the non-illuminated aperture, and a “trial” as the receipt of reward following one or more cued responses. Trials were self-initiated by the mice; therefore, the number of trials per session was open-ended. For each mouse, the light cue was presented in the same aperture during every trial across days. Across mice, cue location (left or right aperture) was randomized. To encourage responding, food rewards were delivered on a FR1 schedule (i.e., fixed ratio 1: reinforcement given after each response) for the first 10 rewards, then on a VR2 schedule (i.e., variable ratio 2: delivery of reinforcement varied but given after 2 responses on average) for the following rewards. Mice were also “primed”: on the first 2 days of training, one food pellet was placed inside both the cued and non-cued aperture. Subsequently, mice were “re-primed” for 1 day following training days on which they completed <5 trials. Mice needed to complete >15 trials within a session, and >75% cued response rate [cued responses/(cued + non-cued responses)], over 5 consecutive days to meet criteria for successful operant acquisition. Immediately following acquisition, mice underwent 3 days of extinction training, where the light cue was presented without reward. Where noted, cued and non-cued responses were normalized to the group average of the last 5 days of acquisition.

Flurothyl seizure kindling and analysis. Independent cohorts of *Ube3a*^{OE} and WT control mice were used for these studies. Briefly, mice were habituated for 1 minute in a lidded 2-L glass chamber prior to infusion of 10% flurothyl (bis-2,2,2-trifluoroethyl ether; Sigma-Aldrich) in 95% ethanol at a rate of 200 μL/minute onto a disk of filter paper (Whatman, Grade 1) suspended at the top of the chamber. Myoclonic (sudden involuntary jerk/shock-like movements involving the face, trunk, and/or limbs) and generalized seizures (clonic-forebrain seizures characterized by clonus of the face and limbs, loss of postural control, rearing, and falling) were carefully monitored. Upon emergence of a generalized seizure, the lid of the chamber was immediately removed, allowing for rapid dissipation of the flurothyl vapors and exposure of the mouse to fresh air. Mice were then returned to their home-cage following

recovery from behavioral seizures. Between trials, the flurothyl chamber was recharged with fresh filter paper, cleaned using 70% ethanol, and thoroughly dried. For kindling, flurothyl exposures were repeated once daily over eight consecutive days (i.e., the induction phase). Mice were then given a 28-day rest period in their home-cages (i.e., the incubation phase), prior to a receiving a final flurothyl re-exposure (i.e., rechallenge). Each flurothyl trial was video-recorded and reviewed to determine latency to the onset of myoclonic and generalized seizures, and seizure severity was scored using a modified Racine scale (17).

LTP. Mice were sacrificed by cervical dislocation under isoflurane anesthesia. Brains were quickly isolated, placed in ice-cold oxygenated (95%) and carbonated (5%) artificial cerebrospinal fluid (ACSF: 120 mM NaCl, 3.5 mM KCl, 2.0 mM $CaCl_2$, 1.3 mM $MgSO_4$, 1.25 mM NaH_2PO_4, 26 mM $NaHCO_3$ and 10 mM D-glucose, pH=7.3). Sagittal slices (400 μm) were cut using a Vibratome and hippocampi were dissected out afterwards. Subsequently, these slices were left to recover in oxygenated and carbonated ACSF for 1.5 hours. Extracellular field recordings were performed by submerging the slices in a recording chamber which was continuously perfused with ACSF (flowrate of 2 mL/minute, temperature was kept at 30 ± 0.5 °C). Stimulating and recording electrodes were placed on the CA3-CA1 Schaffer-collateral afferents and the dendrites of CA1 pyramidal cells in the stratum radiatum, respectively. After the electrodes were put in place, the slices were given 30 minutes of rest before starting the measurements. LTP was evoked with a 100 Hz tetanization burst protocol (1 train of 1 second at 100 Hz), performed at one-third of the maximum field excitatory postsynaptic potential (fEPSP). Theta burst LTP (4 trains of 4 stimuli at 100 Hz, 200 ms apart) was evoked at two-thirds of the slice maximum. The average of the last 10 measurements of the normalized fEPSP slope was used to define the magnitude of LTP.

RNAseq. Cortical and hippocampal tissues were dissected from the brains of P7 WT and *Ube3a*$^{+2}$ mice. RNA was immediately isolated using the QIAGEN RNeasy Mini Kit (QIAGEN, 74104) and stored in -80°C for later use. RNAseq was done in collaboration with the Biomics Department at the Erasmuc MC, Rotterdam. Samples were prepped with the Illumina TruSeq Stranded mRNA Library Prep Kit. Sequencing of the resulting DNA libraries

was done according to the Illumina TruSeq Rapid v2 protocol using the Illumina HiSeq2500 sequencer. Generated read-length was 50 base-pairs. Adapter sequences were trimmed off, and the trimmed reads were mapped against the mouse mGRCm38 reference genome using the HiSat2 tool (version 2.1.0) in R. Gene expression values and count tables were generated using the htseq-count tool (version 0.11.2) in R (RRID:SCR_000432). For subsequent analysis of the RNAseq dataset, the Galaxy web platform was used (RRID:SCR_006281). Count-tables were uploaded to the platform, and the DEseq2 tool was used to assess differential gene expression between WT and *Ube3a*$^{+2}$ mice (18). All RNAseq data discussed here are deposited on the NCBI's Gene Expression Omnibus (GEO) (19) and are publicly available through GEO Series accession number GSE205128 (https://www.ncbi.nlm.nih.gov/geo/query/acc.cgi?acc=GSE205128).

Supplemental references

1. Flora DB. Specifying piecewise latent trajectory models for longitudinal data. *Struct. Equ. Model.* 2008;15(3):513–533.
2. Bimboim HC, Doly J. A rapid alkaline extraction procedure for screening recombinant plasmid DNA. *Nucleic Acids Res.* 1979;7(6):1513–1523.
3. De Vree PJP et al. Targeted sequencing by proximity ligation for comprehensive variant detection and local haplotyping. *Nat Biotechnol.* 2014;32(10):1019–1025.
4. Li H, Durbin R. Fast and accurate long-read alignment with Burrows-Wheeler transform. *Bioinformatics.* 2010;26(5):589–595.
5. Jiang Y Hui et al. Mutation of the Angelman ubiquitin ligase in mice causes increased cytoplasmic p53 and deficits of contextual learning and long-term potentiation. *Neuron.* 1998;21(4):799–811.
6. Wang T et al. Enhanced transmission at the calyx of held synapse in a mouse model for Angelman syndrome. *Front Cell Neurosci.* 2018;11:418.
7. Judson MC et al. GABAergic Neuron-Specific Loss of Ube3a Causes Angelman Syndrome-Like EEG Abnormalities and Enhances Seizure Susceptibility. *Neuron.* 2016;90(1):56–69.
8. Schneider CA et al. NIH Image to ImageJ: 25 years of image analysis. *Nat Methods.* 2012;9(7):671–675.
9. Sonzogni M et al. A behavioral test battery for mouse models of Angelman syndrome: A powerful tool for testing drugs and novel Ube3a mutants. *Mol Autism.* 2018;9:47.
10. Nadler JJ et al. Automated apparatus for quantitation of social approach behaviors in mice. *Genes, Brain and Behav.* 2004;3(5):303–314.
11. Serra I et al. Activated PI3Kδ syndrome, an immunodeficiency disorder, leads to sensorimotor deficits recapitulated in a murine model. *Brain Behav Immun Health.* 2021;18:100377.

12. Schreiber J et al. Mechanisms underlying cognitive deficits in a mouse model for Costello Syndrome are distinct from other RASopathy mouse models. *Scientific Reports* 2017;7(1).
13. Omrani A et al. HCN channels are a novel therapeutic target for cognitive dysfunction in Neurofibromatosis type 1. *Mol Psychiatry.* 2015;(11):1311–1321.
14. Achterberg KG et al. Temporal and region-specific requirements of αCaMKII in spatial and contextual learning. *J Neurosci.* 2014;34(34):11180–11187.
15. Goorden SMI et al. Cognitive deficits in Tsc1+/- mice in the absence of cerebral lesions and seizures. *Ann Neurol.* 2007;62(6):648–655.
16. Sidorov MS et al. Enhanced operant extinction and prefrontal excitability in a mouse model of Angelman syndrome. *J Neurosci.* 2018;38(11):2671–2682.
17. Ferland R. The Repeated Flurothyl Seizure Model in Mice. *Bio-Protocol* 2017;7(11):1–13.
18. Love MI et al. Moderated estimation of fold change and dispersion for RNA-seq data with DESeq2. *Genome Biol.* 2014;15(12):550.
19. Edgar R et al. Gene Expression Omnibus: NCBI gene expression and hybridization array data repository. *Nucleic Acids Res.* 2002;30(1):207–210.
